# Prediction of *mycoplasma hominis* proteins targeting in mitochondria and cytoplasm of host cells and their implication in prostate cancer etiology

**DOI:** 10.18632/oncotarget.8306

**Published:** 2016-03-23

**Authors:** Shahanavaj Khan, Mohammed Zakariah, Christian Rolfo, Lembrechts Robrecht, Sellappan Palaniappan

**Affiliations:** ^1^ Nanomedicine & Biotechnology Research Unit, Department of Pharmaceutics, College of Pharmacy, King Saud University, Riyadh, Saudi Arabia; ^2^ Department of Bioscience, Shri Ram Group of College (SRGC), Muzaffarnagar, India; ^3^ Research Center, College of Computer and Information Science, King Saud University, Riyadh, Saudi Arabia; ^4^ Phase I- Early Clinical Trials Unit, Oncology Department, Antwerp University Hospital, “Centre for Oncological Research (CORE)”, Edegem, Belgium; ^5^ School of Science and Engineeringing, Malaysia University of Science and Technology, Selangor, Malaysia

**Keywords:** M. hominis, database, mitochondria, cytoplasm, prostate cancer

## Abstract

Although the idea of bacteria causing different types of cancer has exploded about century ago, the potential mechanisms of carcinogenesis is still not well established. Many reports showed the involvement of *M. hominis* in the development of prostate cancer, however, mechanistic approach for growth and development of prostate cancer has been poorly understood. In the current study, we predicted *M. hominis* proteins targeting in the mitochondria and cytoplasm of host cells and their implication in prostate cancer. A total of 77 and 320 proteins from *M. hominis* proteome were predicted to target in the mitochondria and cytoplasm of host cells respectively. In particular, various targeted proteins may interfere with normal growth behaviour of host cells, thereby altering the decision of programmed cell death. Furthermore, we investigated possible mechanisms of the mitochondrial and cytoplasmic targeted proteins of *M. hominis* in etiology of prostate cancer by screening the whole proteome.

## INTRODUCTION

Prostate cancer (PC) is second common cause of cancer and considered as sixth leading cause of cancer-associated death in among men globally [1–3]. Chronic inflammation connected with various infections has been identified as a most significant cancer-promoting situation in different types of cancers, including colon cancer, lung cancer, prostate cancer etc [4–6]. Various evidences showed the involvement of bacterial infection in progression and development of various types cancers [7–9]. Although exact mechanisms directing of carcinogenesis are not well explored, there are considering evidences that bacteria can involved in growth and development of many types of cancer. Several suggestions have been done that inflammation due to chronic infections is one of the main factors for etiology of PC [4, 6, 10]. The infection of XMRV (xenotropic retrovirus) was identified to be connected with PC, though only in a small percentage of patients of PC [11]. Thus, the main causes regarding PC-promoting chronic inflammation still need to be identified.

Mycoplasmas are considered as common commensals of the human urogenital microbes which may become pathogenic under particular, relatively unusual conditions [12, 13]. The infection of mycoplasma proposes that it could produce chronic inflammation with pro-cancerous causes due to their chronic nature. The connection of *mycoplasmas* infection with malignancy was first detected in 1960s [14]. *Mycoplasma hominis* (*M. hominis*), together with *Ureaplasma urealyticum* and *M. genitalium* are the key species of mycoplasma identified in urogenital tract of human. *M. hominis* is an important extracellular gram negative bacteria, which may be replicated and colonized intracellularly in progression of prostate cancer [15]. This uncommon colonization nature of *M. hominis* in genital tract including prostate cells may have specific implications in the etiology of prostate cancer. Many studies have explained the significant connection of *Mycoplasma hominis* infection with the growth and development of PC [9, 16, 17]. The infection of mycoplasma potentially linked with the disregulation of normal functioning of cell cycle and programmed cell death during apoptosis [18]. Various constant signals could be transmitted to bacterial infected cells through the interaction of mycoplasmas with the surface receptor of host cells, which may ultimately lead to chronic alteration. Therefore, these enigmatic alterations in the mycoplasma infected cells influence to many biological processes including apoptosis and control of normal cell growth [18, 19].

Although the infection of *M. hominis* is associated with an increased risk of prostate cancer, many other factors are also involved in growth and development of prostate cancer [4, 20]. It was observed that approximately 20% of various type of cancers in human are developed due to chronic inflammatory states and/or chronic infection [4]. Chronic inflammation is one of the important factor for stimulation of highly reactive oxygen species and proinflammatory cytokines, which can directly lead to nitration and chlorination in nucleic acid and different proteins [4, 21]. In addition to chronic inflammation and mutation, different ranges of *M. hominis* cyclomodulins have been also associated with the growth and progression of PC. Cyclomodulins have the capability to alter the normal functioning of various check points during the cell cycle. It has been assumed that cyclomodulins are an etiological factor for the development of PC [18]. Various infectious agents are susceptible for intracellular colonization and replication in host cells, where they can alter the normal control and functioning of infected cells through host sub-cellular targeting of their protein in the host cells such as the mitochondria, golgi apparatus, endoplasmic reticulum, nucleus, plasma membrane (Secretory protein), cytoplasm etc [15, 22]. It is assumed that different proteins of *M. hominis* may be targeted into various organelles of the host cell where these proteins work as an integrated part of the host proteins due to existence of signature sequences and evolutionary relatedness.

Considered together, we hypothesize that infection of *M. hominis* in prostate cells of host tissue may be connected with progression and development of PC through disturbance of metabolism, apoptosis and generation of chronic inflammation. To predict this hypothesis, we have screened out the *M. hominis* proteins targeting mitochondria and cytoplasm of the host cells and explored the potential implicationof *M. hominis* proteins in etiology of PC using various bioinformatics predictors. The results of the current study suggest that the protein targeting of *M. hominis* in mitochondria and cytoplasm of host cells maybe connected with the growth and development of PC and are, therefore, possible targets for improved prevention, detection, management and cure of PC.

## RESULTS

### Selection of *M. hominis* protein database

UniProt is a comprehensive and broad resource for complete protein sequences and annotation data. The whole proteome of *M. hominis* ATCC-27545 strain was selected from UniProt database due to existence of maximum number of proteins (563) with comparison to other available strain which contain only 529 proteins [23].

### Analysis of mitochondrial subcellular localization of *M. hominis* protein sequences

Out of 563 proteins of *M. hominis* (whole proteome), only 77 proteins were predicted to target in the mitochondria of the host cell as per outcome of BaCelLo. It was observed that higher cut-off values of monopartite NLS are connected with higher number of *M. hominis* protein targeting in the host mitochondrial except for cut-off value 8.0–10 although inverse results are seen with bipartite NLS values where the raising cut-off value decreases the over all percentage of protein targeting in the host mitochondria with their respective range except for cut-off value 3–5 (Table 1). It was also seen that increasing molecular weight is associated with decrease protein targeting to the host mitochondria in there respective range except little deviation in molecular weight 60-80 kD. The lower molecular weight (0-20kDa) proteins were found mainly targeting to the mitochondria of host cells (Table 2). Furthermore, no accurate correlation was found between isoelectric point (pI) values and mitochondrial protein targeting (Table 3). The distribution of *M. hominis* proteins targeting in the host mitochondria with various parameters are illustrated in Figure S1, whereas the pattern of all proteins targeting in mitochondria with diverse parameters is illustrated in Figure S2. The Supplementary Material provides detailed information regarding proteins targeted to mitochondria of host cells through prediction analysis (Table S1).

**Table 1:** Computational prediction of *Mycoplasma hominis* proteins targeting to mitochondrion of host cells and their relation with all proteins with similar NLS

NLS	NLS cutoff	Number of proteins targeting Mitochondrion	Total number of proteins in this range	Percentage
**Monopartite NLS**	1.0-2.0	63	490	12.85
	3.0-5.0	8	45	17.77
	7.0-8.0	5	18	27.77
	>8.0	1	10	10
**Bipartite NLS**	1.0-2.0	3	33	09.09
	3.0-5.0	39	246	15.85
	7.0-8.0	34	271	12.54
	>8.0	1	13	07.69

**Table 2:** Computational prediction of *Mycoplasma hominis* proteins targeting to mitochondrion of host cells and their relation with all proteins with similar molecular weight

Molecular Weight	Number of proteins targeting to Mitochondrion	Total number of proteins	Percentage
**0-20 kD**	32	128	25
**20-40 kD**	28	205	13.65
**40-60 kD**	7	109	6.42
**60-80 kD**	9	61	14.75
**>80 kD**	1	60	1.66

**Table 3:** Computational prediction of *Mycoplasma hominis* proteins targeting to mitochondrion of host cells, and their relation with all proteins with similar pI value

Range of pI value	Number of proteins targeting to Mitochondrion	Total number of proteins	Percentage
**3.0-5.0**	0	24	0%
**5.0-6.0**	3	104	2.88%
**6.0-7.0**	3	70	4.28%
**7.0-8.0**	1	35	2.85%
**8.0-9.0**	11	106	10.37%
**9.0-10**	35	182	19.23%
**10.0-11.0**	21	35	60.00%
**11.0-13.0**	3	7	42.85%

### Analysis of cytoplasmic subcellular localization of *M. hominis* protein sequences

Out of 563 proteins of *M. hominis* only 320 proteins were observed to target to the cytoplasm of host cells through *in silico* analysis using BaCelLo predictor. It was found that raising NLS cut-off values of monopartite and bipartite are associated with decreased number of *M. hominis* protein targeting in cytoplasm except cut-off value 7.0-8.0. Largest proteins containing cut-off value 1.0-2.0 were predicted to target in cytoplasm of host cell with monopartite as well bipartite NLS (Table 4).

**Table 4:** Computational prediction of *Mycoplasma hominis* proteins targeting to cytoplasm of host cells and their relation with all proteins with similar NLS

NLS	NLS cutoff	Number of proteins targeting Cytoplasm	Total number of proteins in this range	Percentage
**Monopartite NLS**	1.0-2.0	282	490	57.55
	3.0-5.0	24	45	53.33
	7.0-8.0	10	18	55.55
	>8.0	4	10	40
**Bipartite NLS**	1.0-2.0	20	33	60.6
	3.0-5.0	139	246	56.5
	7.0-8.0	155	271	57.19
	>8.0	6	13	46.15

However no exact correlation was observed between different range of molecular weight and proteins targeting in cytoplasm. Proteins with Molecular weight 40–60 kDa were found mostly target to cytoplasm of host cell (Table 5). Similarly, different range of isoelectric point (pI) did not illustrate accurate pattern for *M. hominis* protein targeting in cytoplasm of host cell (Table 6). The patterns of *M. hominis* protein targeting with various parameters are demonstrated in Figure S3. Moreover, Supplementary Material provides detailed information regarding all proteins targeting to the cytoplasm of host cells through prediction analysis (Table S2).

**Table 5:** Computational prediction of *Mycoplasma hominis* proteins targeting to cytoplasm of host cells and their relation with all proteins with similar molecular weight

Molecular Weight	Number of proteins targeting to Cytoplasm	Total number of proteins	Percentage
**0-20 kD**	64	128	50
**20-40 kD**	118	205	57.56
**40-60 kD**	78	109	71.55
**60-80 kD**	27	61	44.26
**>80 kD**	33	60	55

**Table 6:** Computational prediction of *Mycoplasma hominis* proteins targeting to cytoplasm of host cells and their relation with all proteins with similar pI value

Range of pI value	Number of proteins targeting to Cytoplasm	Total number of proteins	Percentage
**3.0-5.0**	15	24	62.5
**5.0-6.0**	81	104	77.88
**6.0-7.0**	59	70	84.28
**7.0-8.0**	27	35	77.14
**8.0-9.0**	52	106	49.05
**9.0-10**	74	182	40.65
**10.0-11.0**	9	35	25.71
**11.0-13.0**	3	7	42.85

## DISCUSSION

Mycoplasmas are supposed to be the simplest and smallest organisms with self-replicating ability, harbouring 0.3 – 0.8 μm range of diameter. Various pathogens including bacteria expresses diverse effector molecules, able to modify the host transcriptome, epigenome, proteome and metabolome and through different strategies involving targeting of host chromatin, alteration in epigenetic regulators, protein modification and degradation, cell signalling pathways, apoptosis, production of cytokines and growth factor, cytoskeletal rearrangement, phagocytosis etc [24–26]. Therefore, bacteria possess enough capability to alter the normal functioning of regulatory pathways of infected host cells for their growth and survival. *M. hominis* is a gram negative pleomorphic facultative intracellular bacterium associated with a variety of urogenital infections [27]. This facultative bacterium is more tightly connected with host cells. It has been mentioned earlier that mycoplasma has the potential to change the host cell proteome [28].

Moreover, during chronic infection of bacteria including *M. homini*, various proteins of this pathogen will be targeted to different compartments of host cell, which may act as integral part of the host cell. In this condition, it can be supposed that some proteins of *M. humini* can release to different cell organelles including the host cell mitochondria, cytoplasm, nucleus, plasma membrane etc [29]. These migrated proteins can exert diverse effects in the host cell and alter the normal functioning of the effected host cell such as promoting or inhibiting certain essential biological activities which may lead to the growth and development of cancer [30]. Many tools are available for the prediction of subcellular targeting of particular proteins, which are based on different rules and principles including detection of specific motifs through the artificial neural feed-forward network, sequence alignment/similarity search, the self-organizing map (SOM), the support vector machine (SVM), the Hidden-Markov-Model (HMM), etc. Though various experimental high-throughput advance methods have been developed currently for the analysis of proteins localization [31, 32], they are more time taking and less cost effective. Nowadays valuable computational tools are available for predicting subcellular targeting of proteins with promising accuracy and genome-scale due to advancement in bioinformatics, which provide attractive complement to experimental results [33, 34].

NLS Mapper is a useful widget that provides NLS values particularly the importin αβ pathway by predicting NLS scores. cNLS Mapper calculates classical NLS (cNLS) functionality values of particular query sequence of proteins by predicting the total functional contribution of all amino acid residues as per the information based on activity-profiles that are obtained from the systematic residue-replacement predictions in *Saccharomyces cerevisiae*. Prediction of NLS in any protein sequence is necessary for the predicting of their nuclear localization with the maximum cNLS value. Therefore, cNLS mapper is a tool of bioinformatics for the prediction of NLS activity instead of NLS sequence, and their calculated activity demonstrates cytoplasmic localization of different sequences of protein [20, 35, 36].

**Figure 1:**
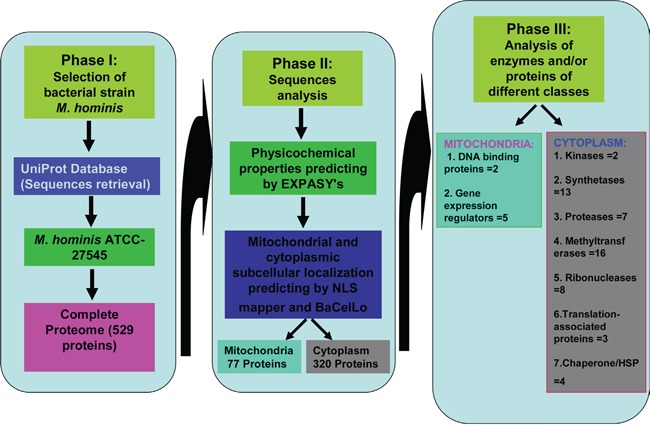
Summary of possible functions of *Mycoplasma hominis* proteins targeted to mitochondria and cytoplasm of the host cells in development of prostate cancer

On the contrary, other bioinformatics predictor BaCelLo utilizes a decision tree which are based on different SVMs in order to evaluate mitochondrial, cytoplasmic, nuclear, and chloroplast targeting of particular proteins [22]. BaCelLo predictor evaluates query sequence of particular protein along with it's C and N termini. The results of BaCelLo predictor generated on the basis of information achieved through available sequences of amino acid and evolutionary alignment. In the current study, we have predicted *M. homini* proteins targeting in cytoplasm and mitochondria of host cell through ExPASy Compute pI/Mw tool, NLS mapper and BaCelLo predictors in order to achieve appropriate idea of mechanism of carcinogenesis of prostate cancer (Figure 1).

### *M. homini* proteins targeting in host mitochondria and their role in prostate cancer

The most essential organelle of the cell after the nucleus are the mitochondria, controlling many biological events such as rate of biosynthetic, bioenergetic status, maintaining the level of cytosolic Ca++, generation of reactive oxygen species (ROS) and potentially involved in intrinsic pathway of apoptosis [28]. Cancerous cells illustrates antagonistic properties towards intrinsic pathways of apoptotic [27, 28]. The symbiotic partnership of bacterium and mitochondria is approximate 2000 million year ago as nuclear–cytosolic organism [37, 38]. However it is not clear accurately what is the possible role of the bacteria and their proteins in development of different types of cancer, bacterial proteins that change the normal functions of mitochondria should attach to mitochondria and/or they must enter to the mitochondria during the course of infection. It is evident that many bacteria dynamically impeded or inhibited the process of programme cell death in host cells for their replication and persistence [4, 6, 22].

The morphology of host cells may be changed during the course of bacterial infection. Cancer cells show alteration in morphology and biochemical properties including lack of surface attachment properties, transformation from normal to round shape properties and increased rate of consumption of glucose for generation of ATP by alteration in glycolysis with high lactic acid fermentation. While lower rate of glucose metabolism is a common event in tricarboxylic acid (TCA) cycle of normal cells, it may be assumed that uncontrolled cancer cells have hindered mitochondrial oxidative phosphorylation due to the difficulty in finding such uncommon observation. Although without any significant evidences, various similar phenomena have been detected in mitochondria of cancerous cells [26, 27]. In the present study, we have evaluated 77 proteins targeting to the mitochondria of host cells from complete proteome of *M. homini* containing 563 proteins. These targeted proteins may have various effects on the normal functioning of mitochondria of host cells.

### Implication of DNA binding proteins and gene expression regulators in prostate cancer etiology

Mitochondria have own genetic material and their specific protein-synthesizing machinery, which are comparable to bacteria. The possible connection of bacterial DNA binding proteins and gene expression regulators with growth and development of different types of cancers have been observed in various studies. Various DNA-binding proteins were analysed to target host cell mitochondria including WhiA family regulator (A0A097NTW0), putative holliday junction resolvase (A0A097NST7), what may effects normal functioning of replication and gene expression in mitochondria (Table 7). DNA-binding protein-WhiA is a distant homolog of eukaryotic LAGLIDADG endonucleases [39]. Various endonucleases which are called as ubiquitous catalysts actively engaged in genomic alteration, DNA rearrangement/mutation, repair and protection by generating cuts into double-stranded DNA. Similarly, bacterial holliday junction resolvase enzyme is involved in recombination and repair events [40]. Although little information is available regarding eukaryotic holliday junction resolvases, the enzyme has showed to be active especially in mitochondria [41, 42]. We can assume that the presence of bacterial DNA-binding proteins may act as possible etiological factor in growth and development of cancer. In the same manner, various gene expression regulators of *M. homini* were predicted to target in host mitochondria (Table 7).

**Table 7:** Possible functions of *M. homini* proteins targeted to mitochondrial of host cells and involvement in growth of prostate cancer

S. No.	*M. homini* host mitochondrial targeted protein and enzymes with accession numbers	Possible functions in cytoplasm	References
1.	**DNA binding proteins**:WhiA family regulator (A0A097NTW0)Putative holliday junction resolvase (A0A097NST7)	**DNA binding proteins**:Many DNA-binding proteins may actas potential factors in growth andprogression of cancer	[39–41]
2.	**Gene expression regulators**:Ribonuclease III (A0A097NTT5)Endonuclease IV (A0A097NT17)Excinuclease ABC (A0A097NSM8)tRNA (guanine-N(1)-)-methyltransferase (A0A097NT75)Ribosomal RNA small subunit methyltransferase I (A0A097NSP3)	**Gene expression regulators**:Different gene expression regulatory involved inDNA repair, breaking ofds RNA which includingribonucleases, endonuclease andmethyltransferase.	[43–46]

Ribonuclease III enzymes are very conserved family of proteins from Bacteria to Eukarya that specifically cleave double-stranded (ds)RNA [43]. These enzymes have a variety of functions, including processing of ribosomal RNA [44]. Bacterial Endonuclease IV is orthologue of APE1 that executes AP site cleavage as APE1 but has different mechanistical and structural active site [45, 46]. Excinuclease, also called as excision endonuclease is a nuclease enzyme that excises a specific number of nucleotides during the process of DNA repair. Therefore, these mitochondria targeted endonuclease and other proteins of *M. homini* have the possibility to interfere with mitochondrial DNA/RNA and may act as potential factors for the growth and development of prostate cancer.

### *M. homini* proteins targeting host cytoplasm and their role in prostate cancer

Many bacteria that cause severe infections in host cells, including humans, have the capability to invade the host cells and to replicate efficiently either in the cytoplasm or in other specific compartments of the infecting cells. Various biochemical pathways in cytoplasm of host cells may alter due to *M. homini* protein targeting in cytoplasm during the course of infection. The present study showed that several proteins of *M. homini* target to the cytoplasm of host cells as described in Supplementary Table (Table S2). Among these cytoplasm targeting proteins, many proteins interferes with host proteins and may alter the normal functioning of translation, nucleotide biosynthesis, nucleotide degradation, mRNA, tRNA and rRNA. In this section, we are focusing on different proteins and their enigmatic involvement in growth and development of prostate cancer.

### Possible effect of *M. homini* proteins on normal biochemical pathways

The result of *M. homini* protein targeting in host cell cytoplasm may be helpful to the researcher for better understanding that how these proteins contribute in lung cancer etiology. Initially, it has been supposed that cancer is a disease of proliferation of cells, current data has proposed that altered metabolism can also be a considerable factor of cancer [47]. The infection of mycoplasma was reported to stimulate nucleoside catabolism in host cell and inhibit the anabolism of nucleoside *in vitro* [48]. The pioneering work of Otto Warburg showed (1920s,) that most of cancerous cells generate energy largely, despite the accessibility of enough oxygen through increase rate of glycolysis and then lactic acid fermentation in the cytoplasm. Although, normal cells produce a low rate of glycolysis comparatively and then pyruvate oxidation in the mitochondria [49, 50]. This phenomenon is called as the Warburg effect. Warburg observed to be a basic difference in the ratio of glycolysis to respiration between normal and cancer cells. He assumed that this aerobic glycolysis occurred due to respiratory injury [51]. Many kinases involved in normal functioning of metabolism and changes in functions of these enzymes may direct to series of pathological condition including cancer [52]. Various kinases are predicted as cytoplasmic targeting enzyme in our prediction analysis (Table 8) TK enzyme has an important role in the “salvage pathway” of DNA synthesis. The altered level of TK detected in different type of cancer including prostate cancer [53, 54]. Theses kinases may alter the normal rate cells metaboloism in host cells. It has been reported that prolonged aerobic glycolysis in particular cancerous cells is connected to activation of different oncogenes or to suppression of tumor suppressor functions [55, 56]. PK transfers the phosphate group to adenosine diphosphate (ADP) from phosphoenolpyruvate (PEP) and controls the last rate-limiting step of glycolysis. PK, an important enzyme in metabolism of cancer cells and alteration in metabolism is connected with growth and development of cancer [57]. Uridylate kinase (UK) is a key enzyme of pyrimidine nucleoside biosynthesis. It has been reported that alter level of expression in uridylate kinase in various type of cancer [58, 59].

**Table 8:** Possible functions of *M. homini* proteins targeted to cytoplasm of host cells and involvement in growth of prostate cancer

S. No.	*M. homini* host cytoplasm targeted protein and enzymes	Possible functions in cytoplasm	References
1.	Thymidine kinase (TK) (A0A097NT70)Pyruvate kinase (PK) (A0A097NTQ0)Uridylate kinase (UK) (A0A097NTS4)Guanylate kinase (GK) (A0A097NTX8)Adenylate kinase (AK) (A0A097NT99)	**Kinases**: Various enzymesof this class potentially alterthe normal functioning/haemostasisof normalbiochemical pathways thatacts as a possible factor forthe growth of cancer.	[53–59]
2.	**Synthetases**:Cysteinyl-tRNA synthetase (A0A097NSM7)Tyrosyl-tRNA synthetase (A0A097NT68) Prolyl/proline-tRNA synthetase (A0A097NTQ3)Asparaginyl-tRNA synthetase (A0A097NTR5)Leucyl-tRNA synthetase (A0A097NTB2)Alanyl-tRNA synthetase (A0A097NSU6) Isoleucyl-tRNA synthetase (A0A097NTD0)Methionyl-tRNA synthetase (A0A097NSU2)Seryl-tRNA synthetase (A0A097NTQ4), Threonyl-tRNA synthetase (A0A097NSP8)Arginyl-tRNA synthetase (A0A097NSL8)Tryptophanyl-tRNA synthetase (A0A097NSN0)Histidyl-tRNA synthetase (A0A097NSZ6).	**Synthetases**: Involvedin ARSs are involved ingrowth and development ofvarious diseases includingvarious neuronal diseases,autoimmune diseases and cancer	[60, 61]
3.	**Proteases**:Methionine aminopeptidase (A0A097NT82), Cytosolaminopeptidase (A0A097NTR6, A0A097NTN2), AminopeptidaseC (A0A097NSH4), ATP-dependent zinc metalloprotease(A0A097NTQ7), Xaa-Pro aminopeptidase (A0A097NT20)ATP-dependent protease Lon (A0A097NTL4)	**Proteases**: Differentproteases are involvedin abnormal proteindegradation that may alterthe normal functioning ofhost cells and direct to the progression ofvarious types of cancer.	[62, 63]
4.	**Methyltransferases**:Putative O-methyltransferase (A0A097NSV1)Serine hydroxymethyltransferase (A0A097NTW7)Putative TrmH family tRNA/rRNA methyltransferase (A0A097NT39)Type I restriction modification system DNA methyltransferase HsdM (A0A097NTD3, A0A097NU11)16S/23S rRNA (Cytidine-2′-O)-methyltransferase TlyA (A0A097NSS7) Ribosomal RNA large subunit methyltransferase H (A0A097NU21), Putative RNA uracil-methyltransferase (A0A097NTV7)Ribosomal RNA small subunit methyltransferase G (A0A097NTG0), DNA modification methylase (A0A097NTD7)tRNA (guanine-N(7)-)-methyltransferase (A0A097NTM7)Ribosomal RNA small subunit methyltransferase (A0A097NT40)DNA methylase (A0A097NSF6)Release factor glutamine methyltransferase (A0A097NSY0)5′-methylthioadenosine/S-adenosylhomocysteine nucleosidase (A0A097NSE0)TrmH family tRNA/rRNA methyltransferase (A0A097NSK0).	**Methyltransferases**: Thisclass of enzymes and/or proteinshave methylation activity that involved indevelopment of varioustypes of cancer	[64–67]
5.	**Ribonucleases**:Ribonuclease III (A0A097NSP1) Ribonuclease R (A0A097NSZ8) Endoribonuclease YbeY (A0A097NTQ5)Ribonuclease HII (A0A097NTG4) Endoribonuclease VapD (A0A097NTX3) mRNA degradation ribonucleases J1 (A0A097NTH2)mRNA degradation ribonucleases J2 (A0A097NTK2)endoribonuclease VapD (A0A097NTX3)	**Ribonucleases**: The enzymes of this class are called as nucleases that involved in breaking of phosphodiester bond of RNA. Many enzymes of nuclease engaged in growth and development of different types of mammalian cancer	[68–74]
6.	**Translation-associated proteins**:50S ribosomal protein L36 (A0A097NT94)L24 (A0A097NT93)30S ribosomal protein S20 (A0A097NSM5)	**Translation-associated proteins**: Many proteinsof this category may effectthe gene expression andinvolved in many conditionincluding cancer.	[75–78]
7.	**Heat shock protein (HSP)/Chaperone**:Chaperone protein DnaJ (A0A097NSW0) Chaperoneprotein ClpB (A0A097NSI6) ClpB related chaperone protein(A0A097NT57)HSP-70 cofactor (A0A097NTP0)	**HSP/Chaperone**: It isreported that alteration inexpression of chaperones/heat shock protein (HSP)in different types of cancerincluding prostate cancer	[79–83]

We have also predicted various synthetases enzymes of *M. homini* targeting to cytoplasm of host cell (Table 8). Various synthetase enzymes are constituent of complex of aminoacyl-tRNA synthetases (ARSs), which potentially engaged in the process of protein synthesis. This is reported that 20 different aminoacyl-tRNA synthetases (ARSs) associate with particular amino acid to their specific tRNAs. The specific ARSs are connected with many diseases situation including various neuronal diseases, autoimmune diseases and cancer [60, 61].

We also predicted a variety of *M. homini* protease targeted to cytoplasm of the host cells (Table 8). Recent studies propose that cytosolic peptidases may be potentially involved as a factor of colorectal cancer and helpful for the identification CRC as a prognosis marker [62]. A high level of expression of Methionine aminopeptidase was reported in human colon cancer cell lines and colorectal cancer tissues [63]. These targeted proteases may be involved in abnormal protein degradation and alter the normal functioning of host cells. In addition to aminopeptidase, various methyltransferases enzymes and proteins also analysed as targeted to cytoplasm of host cell in our current study (Table 8). Many enzymes of methyltransferases class actively involved in site-specific methylation which have important role in cancer therapeutics [64]. It was reported that catechol-O-methyltransferase involves in breast cancer and estrogen-linked endometrial cancers due to their cancer-promoting activities [65, 66]. It was demonstrated that silencing of mitochondrial serine hydroxymethyltransferase inhibits proliferation of cancer cell [67]. In the present study we have predicted *M. homini* serine hydroxymethyltransferase (A0A097NTW7) targeting to cytoplasm of host cells. The rate of carcinogenesis may increase in prostate cell due to targeting of *M. homini* serine hydroxymethyltransferase in cytoplasm of host cell.

### Possible effect of *M. homini* enzymes/proteins in host translation

Translation or protein synthesis is the largely energy-devouring process of the cell. It is a tightly controlled process, involving the coordinated interaction of mRNA, ribosomes, tRNAs and various auxiliary factors. Alteration in normal regulation of translation can be supposed as a potential hallmark of growth and development of cancer which is connected to abnormal proliferation, angiogenesis, cancer energetics and variations in immune response [68–71]. The cytoplasmic targeting of various enzymes and protein of *M. homini* were studded in present study, which have high degree of potential of interaction with various regulatory elements and RNA of host translation machinery. Many reports revealed the involvement of different RNA-binding enzymes in progression and development of cancer [72, 73]. Similarly, various RNA-binding proteins have connected with the growth of cancer through the control of gene expression at the post-transcriptional level [72].

Ribonucleases are a class of nuclease breaking the phosphodiester bond of RNA and involving in various cellular consequences in host cell. It was studied that various ribonucleases engaged in development as well prevention of different types of mammalian cancer. Although the potential involvement of bacterial ribonucleases is not much more cleared in cancer during the course of infection Therefore, involvement of bacterial ribonucleases in progression of cancer requires more research and precise biological corroborations in this direction. In our prediction analysis certain ribonucleases were predicted to target in the cytoplasm of host cell (Table 8). Ribonuclease III (A0A097NSP1) is involved in processing of mRNA, rRNA, and tRNA while ribonuclease R (A0A097NSZ8) contains exoribonuclease II activity. Similarly, endoribonuclease YbeY (A0A097NTQ5) is engaged in rRNA processing whereas and ribonuclease HII (A0A097NTG4) has RNA-DNA hybrid ribonuclease activity. In addition, endoribonuclease VapD (A0A097NTX3) has hydrolase activity wheras mRNA degradation ribonucleases J1 and J2 (A0A097NTH2, A0A097NTK2) exhibited endonuclease activity in *M. homini* (Table 8). It was reported that tRNA 3′ processing endoribonuclease connected with the development of prostate cancer [74]. The cytoplasmic localization of different ribonucleases of *M. homini* in host cell increases more risk of cancer and act as potential factors of prostate cancer etiology.

Furthermore, various studies confirmed the possible association of mRNA-translation-associated proteins in progression and development of cancer [75, 76]. Current researches are forced on translation regulator as therapeutic target in cancer [77, 78]. In this predictive study many ribosomal proteins of *M. homini* were analysed to target the cytoplasm of host cells that can also acts as RNA-binding proteins, which are structural constituent of ribosome and involved in mRNA translation (Table 8). Thinking about the important role of RNA-binding proteins in etiology of cancer and various RNA-binding-proteins of *M. homini* targeted to cytoplasm of host cell, it increases doubt for the enigmatical connection of these predicted *M. homini* proteins in etiology of prostate cancer. Although, this predictive results are also needed as suitable experimental validation prior to final conclusion regarding involvement of cytoplasmic targeting of *M. homini* protein in human cells and their potential role in prostate cancer etiology.

### Possible effect of heat shock proteins of *M. homini* in prostate cancer etiology

Various studies confirm the involvement of alteration in expression of chaperones/heat shock protein (HSP) in different types of cancer including prostate cancer [79–81]. It was confirmed in a report that brain tumor cell lines have significant high levels of HSP [82]. The current study showed that various conserved chaperones and HSP cofactor of *M. homini* were targeting to cytoplasm of the host cells (Table 8). These chaperone and HSP cofactor are involved in proper protein folding in *M. homini* during stress conditions. It was studied that various chaperones are vastly conserved in prokaryotes and eukaryotes, which are linked with the development of different types of cancer [82, 83]. A recent report demonstrated that HSPs are possible therapeutic targets in prostate cancer [84]. Nevertheless, the enigmatical involvement of cytoplasmic targeted *M. homini* chaperons/HSPs in human prostate cancer etiology during bacterial infection requires strong experimental cross-validation prior to ultimate conclusion.

### Conclusions

We proposed several mechanisms controlled through the various proteins of *M. homini*, which alters the normal functioning of various pathways in mitochondria and cytoplasm of host cells. Although the potential association of *M. homini* with the risk of prostate cancer during infection have gained credibility in the past many years, no accurate relations have been successfully developed [9, 17]. Therefore *M. homini* associated prostate cancer etiology needs more laboratories based on investigational research to confirm the involvement of *M. homini* proteins targating to mitochondria and cytoplasm of human cells. These novel predictions support that; *M. homini* infection could be a potential cofactor in carcinogenesis of prostate cancer. Various proteins of *M. homini act as* own components of the mitochondria and cytoplasm in host cells. They disturb homeostasis by initiating unnecessary interaction with different proteins of cytoplasm and mitochondria of host cells through different strategies.

The current surprising and elegant findings connect new threads to the etiology of prostate cancer. The intertwining relationship between *M. homini* targeting proteins and prostate cancer would open new doors of advanced research in the future. Understanding the expanding and interconnected roles of *M. homini* proteins targeting in mitochondria and cytoplasm of host cells would contribute to the development of therapies that target prostate cancer. Advanced research in this direction of bacterial-host protein interaction with in host cell can validate the bacterial regulatory elements responsible for growth and development of prostate cancer and their etiology. Thus, the present research definitely emerges confidence towards explaining the enigmatic role *M. homini* in prostate cancer etiology.

## MATERIALS AND METHODS

### Selection of *M. hominis* protein database

In the present study we revealed that numerous *M. hominis*-derived proteins can transfer to cytoplasm and the mitochondria of the host cells and can involve in the growth and development of PC. The whole proteome of existing bacteria *M. hominis* were utilized for *in silico* analysis of subcellular protein targeting the mitochondria and cytoplasm of host cells and their potential implication in growth and development of PC. The UniProt database was used to predict the proteins of *M. hominis* targeting host cells. This database was generated by the collection of TrEMBL, Swiss-Prot and PIR protein database activities [31]. The UniProt database includes immense information regarding *M. hominis* proteins and their subcellular targeting as mentioned in Swiss-Prot/TrEMBL or PIR-PSD [32–34]. Complete protein sequences of two strains of *M. hominis* (ATCC-23114/PG21 and ATCC-27545) were accessible in the UniProt database [35]. The complete proteome of ATCC-27545 strain of *M. hominis* was used for analysis of mitochondrial and cytoplasmic targeting in human prostate cell using ExPASy Compute pI/Mw tool, Balanced Subcellular localization (BaCelLo) predictor and cNLS mapper (Figure 1). ExPASy Compute pI/Mw tool was used for the prediction of theoretical pI (isoelectric point) and Mw (molecular weight) in protein sequences.

### Prediction of nuclear localization signal (NLS) in *M. hominis* protein sequences

Previously cNLS mapper has used for the analysis of *E. coli* proteins targeting in mitochondria and cytoplasm of the host cells and their important role in development of colorectal cancer [85]. We utilized cNLS mapper for the analysis of nuclear localization signal (NLS) in the query protein sequence of *M. hominis* [86]. The analysis of NLS is essential to observe nuclear targeting of specific proteins, the potential cut-off value of NLS can also be helpful for possible targeting of particular query protein in cytoplasm of host cell. The *M. hominis* proteins with specific cut-off values 1-2, 5-3, 7-8 and 8-10, were analysed as absolute target to the cytoplasm, both cytoplasm and nucleus, partly target to the nucleus and particularly target to the nucleus of host cell respectively, as explained in existing literature of cNLS mapper. Both monopartite as well as bipartite NLSs were analysed in whole proteins of *M. hominis* for eukaryotic cell. The specific ranges of cut-off values of NLS were predicted in *M. hominis* proteins, in order to analyse mitochondrial and cytoplasmic targeting in host cell. Proteins showing intermediary NLS cut-off values were included in an appropriate range as per their specific cut-off value (i.e., the cut-off value 5.4 or below was included in 3-5 whereas 6.5 or higher cut-off value were included in 7-8.).

### Prediction of subcellular localization in *M. hominis* protein sequences

Recently BaCelLo (**Ba**lanced Sub**cel**lular **Lo**calization) predictor has used for the prediction of *E. coli* proteins targeting in mitochondria and cytoplasm of the host cells and their implication in development of colorectal cancer [85]. Subcellular targeting of *M. hominis* proteins in the host cell was analysed by BaCelLo. BaCeILo predictor was utilized to predict mitochondrial and cytoplasmic localization of *M. hominis* proteins in eukaryotic host cell. BaCelLo, a useful predictor of system biology is based on different support vector machines (SVM). This bioinformatics predictor systematized into a decision tree for the analysis of protein subcellular targeting in five different organelles including mitochondrion, cytoplasm, chloroplast, secretory (Plasma membrane), and nucleus [36]. This predictor can analyse targeting of particular protein in three kingdoms (Animals, Plants and Fungi). In the current study, we predicted *M. hominis* protein subcellular targeting within the animal kingdom.

### Data availability

Raw data of sequences and processed data are available at the UniProt database under Mycoplasma hominis ATCC 27545 strain genome accession number CP009652 [23].

## SUPPLEMENTARY MATERIALS FIGURES AND TABLES







## Abbreviations

*M. hominis*: *Mycoplasma hominis*
NLS: Nuclear localization signal
BaCelLo: Balanced Subcellular Localization
PC: Prostate Cancer
pI: Isoelectric point
TK: Thymidine kinase
PK: pyruvate kinase
UK: Uridylate kinase
GK: Guanylate kinase
AK: Adenylate kinase
