# Supplementary material for: Prediction of mycoplasma hominis proteins targeting in mitochondria and cytoplasm of host cells and their implication in prostate cancer etiology
Source: Oncotarget. 2016 Mar 23;8(19):30830–43. doi: 10.18632/oncotarget.8306 (PMC5458171; doi:10.18632/oncotarget.8306)
Supplement: Supplementary file 1 [file oncotarget-08-30830-s001.pdf]

SUPPLEMENTARY FIGURES & TABLES

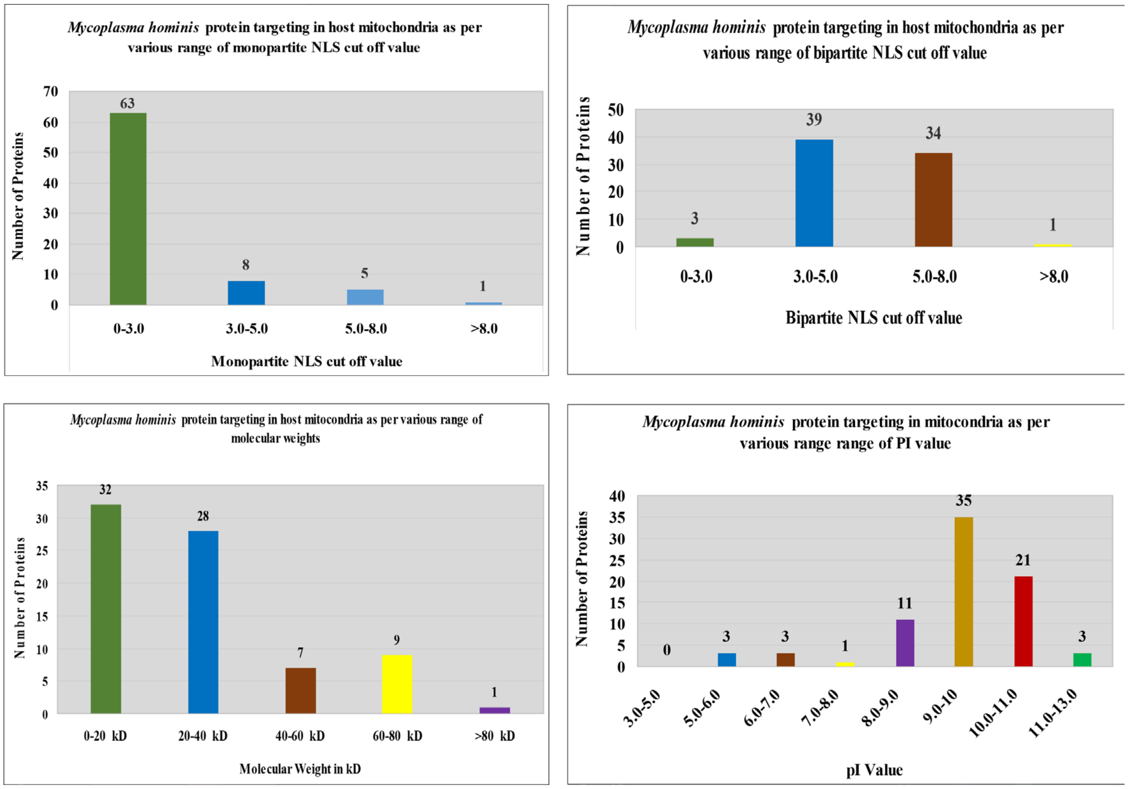

Supplementary Figure S1: Analysis of various parameters during *Mycoplasma hominis* proteins targeting in mitochondria of host cells.

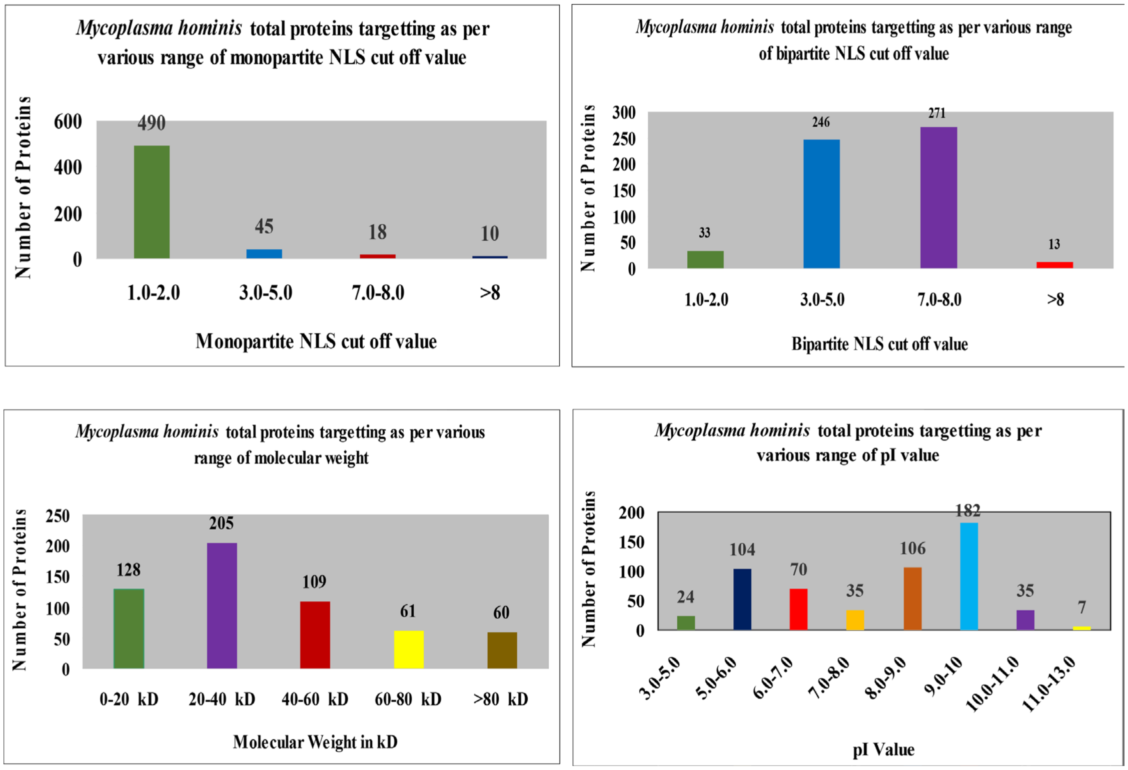

Supplementary Figure S2: Analysis of various parameters during *Mycoplasma hominis* all proteins targeting in host cells.

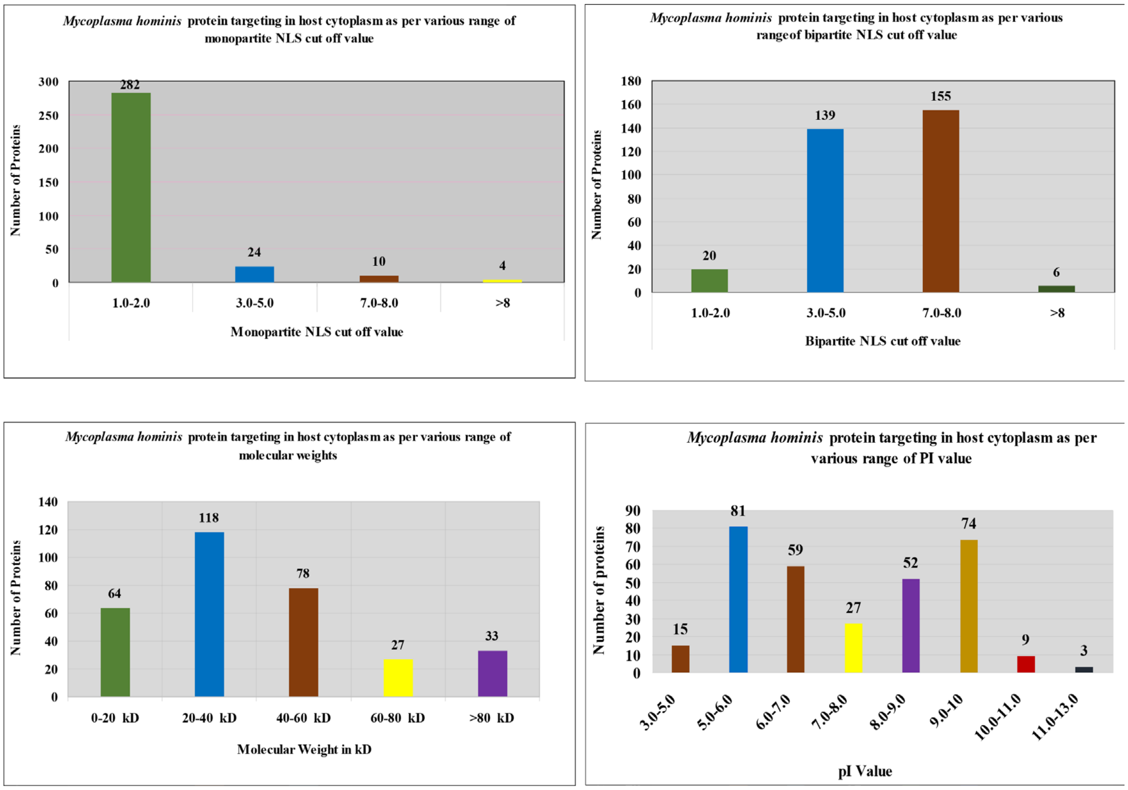

Supplementary Figure S3: Analysis of various parameters during *Mycoplasma hominis* proteins targeting in cytoplasm of host cells.

Supplementary Table S1: Prediction of *M. hominis* proteins targeting in mitochondria of eukaryotic host cells

See Supplementary File 1

Supplementary Table S2: Prediction of *M. hominis* proteins targeting in cytoplasm of eukaryotic host cells

See Supplementary File 2
