# Supplementary material for: Prediction of mycoplasma hominis proteins targeting in mitochondria and cytoplasm of host cells and their implication in prostate cancer etiology
Source: Oncotarget. 2016 Mar 23;8(19):30830–43. doi: 10.18632/oncotarget.8306 (PMC5458171; doi:10.18632/oncotarget.8306)
Supplement: Supplementary file 2 [file oncotarget-08-30830-s002.doc]

**Table S1:** Prediction of *M. hominis* proteins targeting in mitochondria of *M. hominis* proteins in eukaryotic host cells and their function. The proteins details were taken from UniProt database.

| **S.No**. | **Accession number** | **Protein Name** | **Function in bacteria (Gene Ontology)** | **Protein Existence** | **pI** | **Mol. Wt.** | **NLS Mapper**  **Monopartite Bipartite** | | **BaCeILo** |
| --- | --- | --- | --- | --- | --- | --- | --- | --- | --- |
| 1. 417 | A0A097NT02 | Uncharacterized protein | Unknown | Protein predicted | 10.61 | 6793 | 0 | 2.9 | Mitochondrion |
| 1. 68 | A0A097NSV0 | 50S ribosomal protein L35 | Structural constituent of ribosome,translation | Protein inferred from homology | 11.77 | 7089 | 0 | 7.2 | Mitochondrion |
| 1. 124 | A0A097NSH1 | 50S ribosomal protein L28 | Structural constituent of ribosome, translation | Protein inferred from homology | 11.17 | 7112 | 0 | 4.7 | Mitochondrion |
| 1. 391 | A0A097NTA6 | 30S ribosomal protein S14 type Z | rRNA binding, Structural constituent of ribosome, zinc ion binding, translation | Protein inferred from homology | 10.88 | 7202 | 5.5 | 5.2 | Mitochondrion |
| 1. 413 | A0A097NT89 | 50S ribosomal protein L32 | Structural constituent of ribosome, translation | Protein inferred from homology | 10.2 | 7467 | 0 | 5.2 | Mitochondrion |
| 1. 170 | A0A097NTN4 | Preprotein translocase subunit SecE | P-P-bond-hydrolysis-driven protein transmembrane transporter activity,protein secretion | Protein Predicted | 10.66 | 9238 | 0 | 4.9 | Mitochondrion |
| 1. 353 | A0A097NT81 | 50S ribosomal protein L27 | Structural constituent of ribosome, translation | Protein inferred from homology | 10.53 | 9474 | 0 | 2.1 | Mitochondrion |
| 1. 29 | A0A097NT74 | 30S ribosomal protein S16 | Structural constituent of ribosome, translation | Protein inferred from homology | 9.82 | 9809 | 0 | 2.3 | Mitochondrion |
| 1. 199 | A0A097NTA1 | 30S ribosomal protein S19 | rRNA binding, translation, Structural constituent of ribosome | Protein inferred from homology | 10.14 | 10321 | 0 | 5.1 | Mitochondrion |
| 1. 238 | A0A097NT96 | 30S ribosomal protein S17 | rRNA binding, Structural constituent of ribosome, translation | Protein inferred from homology | 9.99 | 10832 | 3 | 4.9 | Mitochondrion |
| 1. 440 | A0A097NST4 | 30S ribosomal protein S18 | rRNA binding, Structural constituent of ribosome, translation | Protein inferred from homology | 10.2 | 11372 | 6.5 | 3.8 | Mitochondrion |
| 1. 197 | A0A097NT88 | 50S ribosomal protein L18 | rRNA binding, translation | Protein inferred from homology | 10.09 | 12843 | 7 | 4.5 | Mitochondrion |
| 1. 266 | A0A097NT95 | 30S ribosomal protein S11 | rRNA binding, Structural constituent of ribosome, translation | Protein inferred from homologyi | 10.27 | 13105 | 0 | 5.2 | Mitochondrion |
| 1. 414 | A0A097NTB1 | 50S ribosomal protein L14 | rRNA binding, Structural constituent of ribosome, translation, | Protein inferred from homology | 10 | 13313 | 0 | 4.1 | Mitochondrion |
| 1. 449 | A0A097NSS5 | 50S ribosomal protein L20 | rRNA binding, Structural constituent of ribosome, ribosomal large subunit assembly, translation | Protein inferred from homology | 11.4 | 13732 | 0 | 6.7 | Mitochondrion |
| 1. 384 | A0A097NT62 | 50S ribosomal protein L19 | Structural constituent of ribosome, translation | Protein inferred from homology | 10.12 | 13883 | 0 | 3.9 | Mitochondrion |
| 1. 82 | A0A097NT92 | 50S ribosomal protein L17 | Structural constituent of ribosome, translation | Protein inferred from homology | 10.43 | 13933 | 0 | 4.4 | Mitochondrion |
| 1. 137 | A0A097NT83 | 30S ribosomal protein S13 | rRNA binding, tRNA binding, translation | Protein inferred from homology | 10.56 | 14013 | 5 | 3.5 | Mitochondrion |
| 1. 14 | A0A097NSZ1 | Uncharacterized protein | Unknown | protein predicted | 9.72 | 14133 | 0 | 6.1 | Mitochondrion |
| 1. 132 | A0A097NTB7 | 50S ribosomal protein L22 | rRNA binding, translation | Protein inferred from homology | 10.32 | 14472 | 0 | 4.6 | Mitochondrion |
| 1. 422 | A0A097NT33 | 30S ribosomal protein S9 | Structural constituent of ribosome, translation | Protein inferred from homology | 10.69 | 14619 | 0 | 2.7 | Mitochondrion |
| 1. 139 | A0A097NT91 | 30S ribosomal protein S8 | rRNA binding, translation | Protein inferred from homology | 9.89 | 15292 | 4.5 | 4.1 | Mitochondrion |
| 1. 374 | A0A097NTA0 | 50S ribosomal protein L15 | rRNA binding, Structural constituent of ribosome, translation | Protein inferred from homology | 10.66 | 15701 | 4 | 6.3 | Mitochondrion |
| 1. 290 | A0A097NSQ1 | 50S ribosomal protein L11 | large ribosomal subunit rRNA binding, Structural constituent of ribosome, translation | Protein inferred from homologyi | 9.59 | 15939 | 3 | 3 | Mitochondrion |
| 1. 245 | A0A097NT26 | 50S ribosomal protein L13 | Structural constituent of ribosome,translation | Protein inferred from homology | 9.92 | 16188 | 0 | 5.5 | Mitochondrion |
| 1. 371 | A0A097NST7 | Putative Holliday junction resolvase (EC 3.1.-.-) | nuclease activity, nucleic acid binding, DNA recombination, DNA repair | Protein inferred from homology | 8.95 | 16374 | 0 | 3.4 | Mitochondrion |
| 1. 456 | A0A097NTB3 | 50S ribosomal protein L16 | rRNA binding, Structural constituent of ribosome, tRNA binding, translation | Protein inferred from homology | 10.44 | 16767 | 0 | 3.2 | Mitochondrion |
| 1. 426 | A0A097NSW2 | 30S ribosomal protein S7 | rRNA binding, Structural constituent of ribosome, tRNA binding, translation | Protein inferred from homology | 9.88 | 17754 | 0 | 6.1 | Mitochondrion |
| 1. 291 | A0A097NTQ2 | Uncharacterized protein | Unknown | Protein predicted | 9.48 | 18621 | 0 | 4.9 | Mitochondrion |
| 1. 36 | A0A097NSM6 | DUTPase | Unknown | Protein Predicted | 9.15 | 19049 | 0 | 7.1 | Mitochondrion |
| 1. 451 | A0A097NTA5 | 50S ribosomal protein L6 | rRNA binding, Structural constituent of ribosome, translation | Protein inferred from homology | 9.65 | 19787 | 0 | 3.6 | Mitochondrion |
| 1. 484 | A0A097NSU7 | Holliday junction resolvase RecU (EC 3.1.22.-) (Recombination protein U homolog) | endonuclease activity, magnesium ion binding, nucleic acid binding, chromosome segregation, DNA recombination, DNA repair | Protein inferred from homology | 9.68 | 19911 | 0 | 4.7 | Mitochondrion |
| 1. 341 | A0A097NT27 | Putative tRNA (cytidine(34)-2'-O)-methyltransferase (EC 2.1.1.207) (tRNA (cytidine/uridine-2'-O-)-methyltransferase) | RNA binding, S-adenosylmethionine-dependent methyltransferase activity, tRNA methyltransferase activity, RNA processing | Protein inferred from homologyi | 8.37 | 20665 | 3 | 5.1 | Mitochondrion |
| 1. 133 | A0A097NT45 | Membrane protein | Unknown | Protein Predicted | 10.47 | 20769 | 0 | 2.5 | Mitochondrion |
| 1. 104 | A0A097NTA8 | 50S ribosomal protein L5 | rRNA binding, tRNA binding, translation | Protein inferred from homology | 9.83 | 21027 | 0 | 2.9 | Mitochondrion |
| 1. 329 | A0A097NTU3 | Ribosomal RNA small subunit methyltransferase D (EC 2.1.1.171) | 16S rRNA (guanine(966)-N(2))-methyltransferase activity, nucleic acid binding | Protein predicted | 8.48 | 21261 | 0 | 4.5 | Mitochondrion |
| 1. 387 | A0A097NSP7 | 30S ribosomal protein S4 | rRNA binding, Structural constituent of ribosome, translation | Protein inferred from homology | 10.08 | 22867 | 4.5 | 5.6 | Mitochondrion |
| 1. 436 | A0A097NT75 | tRNA (guanine-N(1)-)-methyltransferase (EC 2.1.1.228) (M1G-methyltransferase) (tRNA [GM37] methyltransferase) | tRNA (guanine(37)-N(1))-methyltransferase activity | Protein inferred from homology | 8.87 | 25067 | 0 | 4.5 | Mitochondrion |
| 1. 2 | A0A097NTG8 | Uncharacterized protein | unknown | Protein predicted | 9.67 | 25,245 | 0 | 4.2 | Mitochondrion |
| 1. 389 | A0A097NTT5 | Ribonuclease 3 (EC 3.1.26.3) (Ribonuclease III) | metal ion binding, ribonuclease III activity, rRNA binding, mRNA processing, rRNA catabolic process, rRNA processing, tRNA processing | Protein inferred from homology | 9.09 | 26424 | 0 | 3.5 | Mitochondrion |
| 1. 249 | A0A097NSP3 | Ribosomal RNA small subunit methyltransferase I (EC 2.1.1.198) (16S rRNA 2'-O-ribose C1402 methyltransferase) (rRNA (cytidine-2'-O-)-methyltransferase RsmI) | rRNA (cytosine-2'-O-)-methyltransferase activity, enzyme-directed rRNA 2'-O-methylation | Protein inferred from homology | 8.76 | 27014 | 0 | 3.4 | Mitochondrion |
| 1. 360 | A0A097NT55 | Triosephosphate isomerase (TIM) (EC 5.3.1.1) (Triose-phosphate isomerase) | triose-phosphate isomerase activity, gluconeogenesis, glycolytic process,pentose-phosphate shunt | Protein inferred from homology | 6.2 | 27486 | 0 | 8.3 | Mitochondrion |
| 1. 365 | A0A097NSV7 | NH(3)-dependent NAD(+) synthetase (EC 6.3.1.5) | ATP binding, NAD+ synthase (glutamine-hydrolyzing) activity, NAD+ synthase activity,NAD biosynthetic process | Protein inferred from homology | 9.38 | 28847 | 2 | 4.2 | Mitochondrion |
| 1. 34 | A0A097NTC2 | 50S ribosomal protein L3 | rRNA binding, Structural constituent of ribosome, translation | Protein inferred from homology | 9.68 | 28935 | 0 | 4.3 | Mitochondrion |
| 1. 64 | A0A097NSH6 | Ribosomal RNA small subunit methyltransferase A (EC 2.1.1.182) (16S rRNA (adenine(1518)-N(6)/adenine(1519)-N(6))-dimethyltransferase) (16S rRNA dimethyladenosine transferase) (16S rRNA dimethylase) (S-adenosylmethionine-6-N', N'-adenosyl(rRNA) dimethyltransferase) | 16S rRNA (adenine(1518)-N(6)/adenine(1519)-N(6))-dimethyltransferase activity, RNA binding | Protein inferred from homology | 9.12 | 29499 | 0 | 5.5 | Mitochondrion |
| 1. 492 | A0A097NU08 | Uncharacterized protein | Unknown | Protein predicted | 9.49 | 29548 | 0 | 7 | Mitochondrion |
| 1. 517 | A0A097NT03 | Membrane protein | Unknown | Protein predicted | 9.43 | 30878 | 2.5 | 6.3 | Mitochondrion |
| 1. 536 | A0A097NTF7 | Lipoprotein | Unknown | Protein predicted | 9.39 | 31789 | 9 | 3.5 | Mitochondrion |
| 1. 363 | A0A097NTW0 | WhiA family transcriptional regulator | DNA binding, endonuclease activity, regulation of sporulation, regulation of transcription, DNA-templated, transcription, DNA-templated | Protein predicted | 9.37 | 32210 | 0 | 3.9 | Mitochondrion |
| 1. 188 | A0A097NT17 | Probable endonuclease 4 (EC 3.1.21.2) (Endodeoxyribonuclease IV) (Endonuclease IV) | deoxyribonuclease IV (phage-T4-induced) activity, zinc ion binding, DNA repair | Protein inferred from homology | 6.71 | 32211 | 0 | 3.8 | Mitochondrion |
| 1. 283 | A0A097NTR2 | Membrane protein | transporter activity | Protein predicted | 10.19 | 33456 | 4 | 3.2 | Mitochondrion |
| 1. 373 | A0A097NTU4 | GTPase Era | GTPase activity, GTP binding, small ribosomal subunit rRNA binding, ribosomal small subunit biogenesis | Protein inferred from homology | 5.77 | 33876 | 0 | 4.5 | Mitochondrion |
| 1. 110 | A0A097NTF0 | Prolipoprotein diacylglyceryl transferase (EC 2.4.99.-) | phosphatidylglycerol-prolipoprotein diacylglyceryl transferase activity, lipoprotein biosynthetic process | Protein inferred from homology | 9.56 | 35944 | 0 | 3.8 | Mitochondrion |
| 1. 557 | A0A097NSW7 | Uncharacterized protein | Unknown | Protein predicted | 9.89 | 36836 | 0 | 2.7 | Mitochondrion |
| 1. 378 | A0A097NSP2 | ABC transporter permease | transport | Protein inferred from homology | 9.92 | 36927 | 0 | 3.4 | Mitochondrion |
| 1. 59 | A0A097NTM8 | tRNA(Ile)-lysidine synthase (EC 6.3.4.19) (tRNA(Ile)-2-lysyl-cytidine synthase) (tRNA(Ile)-lysidine synthetase) | ATP binding, tRNA modification, | Protein inferred from homology | 8.87 | 37260 | 0 | 5.6 | Mitochondrion |
| 1. 401 | A0A097NSN1 | ABC transporter permease | transport | Protein inferred from homology | 10.47 | 37978 | 2 | 3 | Mitochondrion |
| 1. 563 | A0A097NSI4 | Uncharacterized protein | Unknown | Protein predicted | 9.66 | 38283 | 0 | 3.3 | Mitochondrion |
| 1. 282 | A0A097NU05 | Phosphatase (EC 3.1.3.10) | acid phosphatase activity,glucose-1-phosphatase activity | Protein predicted | 8.73 | 38452 | 0 | 3.4 | Mitochondrion |
| 1. 254 | A0A097NTU9 | Putative glycosyltransferase | transferase activity | Protein predicted | 8.43 | 39729 | 0 | 5.1 | Mitochondrion |
| 1. 528 | A0A097NSW4 | Membrane protein | Unknown | Protein predicted | 9.67 | 40369 | 0 | 6.9 | Mitochondrion |
| 1. 510 | A0A097NSW3 | Uncharacterized protein | Unknown | Protein predicted | 8.79 | 45421 | 0 | 3.8 | Mitochondrion |
| 1. 372 | A0A097NTS3 | Glutamate--tRNA ligase (EC 6.1.1.17) (Glutamyl-tRNA synthetase) | ATP binding, glutamate-tRNA ligase activity, tRNA binding, glutamyl-tRNA aminoacylation | Protein inferred from homology | 6.04 | 53909 | 0 | 3.5 | Mitochondrion |
| 1. 349 | A0A097NT10 | ComEC/Rec2-related protein | Unknown | Protein predicted | 9.65 | 54059 | 0 | 4.4 | Mitochondrion |
| 1. 317 | A0A097NTL6 | 2,3-bisphosphoglycerate-independent phosphoglycerate mutase (BPG-independent PGAM) (Phosphoglyceromutase) (iPGM) (EC 5.4.2.12) | 2,3-bisphosphoglycerate-independent phosphoglycerate mutase activity, manganese ion binding, glucose catabolic process, glycolytic process | Protein inferred from homologyi | 5.99 | 56189 | 0 | 4.3 | Mitochondrion |
| 1. 393 | A0A097NSK2 | Uncharacterized protein | Unknown | Protein predicted | 9.07 | 58325 | 0 | 4.7 | Mitochondrion |
| 1. 418 | A0A097NSJ5 | Potassium transporter KtrB | cation transmembrane transporter activity | Protein predicted | 9.6 | 58434 | 0 | 3 | Mitochondrion |
| 1. 160 | A0A097NTS5 | MATE family protein | antiporter activity, membrane | Protein Predicted | 9.67 | 63281 | 0 | 5.7 | Mitochondrion |
| 1. 232 | A0A097NTJ8 | ABC transporter permease | Transport and permease activity | Protein inferred from homology | 9.86 | 65590 | 0 | 2.9 | Mitochondrion |
| 1. 412 | A0A097NSM8 | Excinuclease ABC subunit C | nuclease activity, DNA repair, SOS response | Protein predicted | 9.22 | 66962 | 0 | 4.4 | Mitochondrion |
| 1. 217 | A0A097NT36 | ABC transporter ATP-binding/permease protein | ATPase activity, coupled to transmembrane movement of substances, ATP binding | Protein inferred from homology | 9.26 | 68449 | 0 | 6.7 | Mitochondrion |
| 1. 174 | A0A097NSX9 | Transketolase (EC 2.2.1.1) | transketolase activity | Protein Predicted | 8.26 | 69472 | 0 | 5 | Mitochondrion |
| 1. 455 | A0A097NTY7 | Putative ABC transporter permease | Unknown | Protein predicted | 9.3 | 70629 | 0 | 5.2 | Mitochondrion |
| 1. 453 | A0A097NSL4 | Uncharacterized protein | Unknown | Protein predicted | 9.24 | 73146 | 6 | 4.7 | Mitochondrion |
| 1. 542 | A0A097NTY5 | Lipoprotein | Unknown | Protein predicted | 8.62 | 74960 | 0 | 6.1 | Mitochondrion |
| 1. 311 | A0A097NTN3 | LMP related protein | Unknown | Protein predicted | 7.95 | 78272 | 3 | 4.6 | Mitochondrion |
| 1. 81 | A0A097NTI5 | P120' protein | Unknown | Protein Predicted | 5.69 | 103639 | 0 | 6.7 | Mitochondrion |
