# Supplementary material for: Prediction of mycoplasma hominis proteins targeting in mitochondria and cytoplasm of host cells and their implication in prostate cancer etiology
Source: Oncotarget. 2016 Mar 23;8(19):30830–43. doi: 10.18632/oncotarget.8306 (PMC5458171; doi:10.18632/oncotarget.8306)
Supplement: Supplementary file 3 [file oncotarget-08-30830-s003.doc]

**Table S2**: Prediction of *M. hominis* proteins targeting in cytoplasm of eukaryotic host cells and their function. The proteins details were taken from UniProt database.

| S.No. | **Accession number** | **Protein Name** | **Function in bacteria (Gene Ontology)** | Protein Existence | **pI** | **Mol Wt** | **NLS Mapper**  **Monopartite Bipartite** | | **BaCeILo** |
| --- | --- | --- | --- | --- | --- | --- | --- | --- | --- |
| 1. 321 | A0A097NTS4 | Uridylate kinase (UK) (EC 2.7.4.22) (Uridine monophosphate kinase) | ATP binding, UMP kinase activity, 'de novo' CTP biosynthetic process, | Protein inferred from homologyi | 5.87 | 27063 | 0 | 0 | Cytoplasm |
| 1. 200 | A0A097NT94 | 50S ribosomal protein L36 | Structural constituent of ribosome, translation | Protein inferred from homology | 11.5 | 4341 | 0 | 3.7 | Cytoplasm |
| 1. 506 | A0A097NU02 | Uncharacterized protein | Unknown | Protein predicted | 11.65 | 4369 | 4 | 2.8 | Cytoplasm |
| 1. 346 | A0A097NSH0 | 50S ribosomal protein L34 | Structural constituent of ribosome, translation | Protein inferred from homologyi | 11.91 | 5687 | 11.5 | 5.5 | Cytoplasm |
| 1. 343 | A0A097NT18 | 50S ribosomal protein L33 | Structural constituent of ribosome, translation | Protein inferred from homologyi | 9.58 | 6009 | 3.5 | 2.9 | Cytoplasm |
| 1. 495 | A0A097NSZ0 | Uncharacterized protein | Unknown | Protein predicted | 9.08 | 6579 | 0 | 2.8 | Cytoplasm |
| 1. 544 | A0A097NSL7 | Uncharacterized protein | Unknown | Protein predicted | 10 | 7565 | 0 | 3.2 | Cytoplasm |
| 1. 26 | A0A097NTB0 | 50S ribosomal protein L29 | Structural constituent of ribosome, translation | Protein inferred from homology | 9.63 | 7586 | 0 | 3.3 | Cytoplasm |
| 1. 493 | A0A097NSW6 | Uncharacterized protein | Unknown | Protein predicted | 10.01 | 7711 | 0 | 2.5 | Cytoplasm |
| 1. 277 | A0A097NT98 | Translation initiation factor IF-1 | translation initiation factor activity | Protein inferred from homologyi | 9.63 | 7990 | 0 | 3.4 | Cytoplasm |
| 1. 549 | A0A097NTW6 | Uncharacterized protein | Unknown | Protein predicted | 10.58 | 8532 | 0 | 5.7 | Cytoplasm |
| 1. 69 | A0A097NTV6 | Uncharacterized protein | Unknown | Protein Predicted | 4.85 | 8539 | 0 | 4.6 | Cytoplasm |
| 1. 87 | A0A097NSX4 | Acyl carrier protein | Unknown | Protein Predicted | 4.9 | 8614 | 0 | 2 | Cytoplasm |
| 1. 488 | A0A097NTC1 | Uncharacterized protein | Unknown | Protein predicted | 10.13 | 8648 | 0 | 3.7 | Cytoplasm |
| 1. 180 | A0A097NTW1 | Putative DNA-binding protein | DNA binding, sigma factor activity, DNA-templated transcription, initiation | Protein Predicted | 5.33 | 8652 | 0 | 2.9 | Cytoplasm |
| 1. 74 | A0A097NSM5 | 30S ribosomal protein S20 | rRNA binding, translation | Protein inferred from homology | 10.43 | 9391 | 3.5 | 3.5 | Cytoplasm |
| 1. 400 | A0A097NT84 | Uncharacterized protein | Unknown | Protein predicted | 9.89 | 10094 | 0 | 4.4 | Cytoplasm |
| 1. 505 | A0A097NSJ2 | Uncharacterized protein | Unknown | Protein predicted | 5.46 | 10243 | 0 | 3.5 | Cytoplasm |
| 1. 98 | A0A097NSI2 | F0F1 ATP synthase subunit C | hydrogen ion transmembrane transporter activity, ATP hydrolysis coupled proton transport | Protein inferred from homology | 6.09 | 10312 | 0 | 0 | Cytoplasm |
| 1. 408 | A0A097NSW8 | DNA-binding protein HU | DNA binding | Protein inferred from homology | 9.52 | 10585 | 0 | 4.1 | Cytoplasm |
| 1. 50 | A0A097NT66 | 50S ribosomal protein L21 | rRNA binding, translation | Protein inferred from homology | 9.76 | 11219 | 4 | 6 | Cytoplasm |
| 1. 138 | A0A097NSM0 | Nucleoid-associated protein MLBD4_00515 | DNA binding | Protein inferred from homology | 4.58 | 11367 | 0 | 2.4 | Cytoplasm |
| 1. 134 | A0A097NSG8 | Glutamyl-tRNA(Gln) amidotransferase subunit C | transferase activity, regulation of translational fidelity | Protein Predicted | 4.58 | 11739 | 0 | 3.1 | Cytoplasm |
| 1. 136 | A0A097NTL8 | Thioredoxin | protein disulfide oxidoreductase activity, cell redox homeostasis | Protein inferred from homology | 4.73 | 11751 | 0 | 2.1 | Cytoplasm |
| 1. 159 | A0A097NSG3 | Holo-ACP synthase | holo-[acyl-carrier-protein] synthase activity, magnesium ion binding, fatty acid biosynthetic process, macromolecule biosynthetic process | Protein Predicted | 8.86 | 11876 | 0 | 3.6 | Cytoplasm |
| 1. 135 | A0A097NTY4 | YlxR family nucleic-acid-binding protein | Unknown | Protein Predicted | 9.49 | 11954 | 0 | 6.1 | Cytoplasm |
| 1. 16 | A0A097NTX3 | Endoribonuclease VapD (EC 3.1.-.-) | hydrolase activity | Protein predicted | 5.53 | 12230 | 0 | 2 | Cytoplasm |
| 1. 284 | A0A097NTI3 | HIT-like protein | hydrolase activity | Protein predicted | 6.18 | 12244 | 0 | 3.3 | Cytoplasm |
| 1. 339 | A0A097NT93 | 50S ribosomal protein L24 | rRNA binding, Structural constituent of ribosome, ,translation | Protein inferred from homologyi | 10.27 | 12434 | 0 | 4 | Cytoplasm |
| 1. 441 | A0A097NSU3 | Uncharacterized protein | Unknown | Protein predicted | 7.73 | 12890 | 0 | 5.9 | Cytoplasm |
| 1. 171 | A0A097NTJ5 | S1 RNA binding domain protein | nucleic acid binding | Protein Predicted | 5.84 | 13040 | 0 | 3 | Cytoplasm |
| 1. 310 | A0A097NT90 | 50S ribosomal protein L7/L12 | Structural constituent of ribosome, translation | Protein inferred from homologyi | 4.93 | 13194 | 0 | 2.5 | Cytoplasm |
| 1. 107 | A0A097NSR3 | IMPACT family member YigZ | Unknown | Protein Predicted | 9.52 | 13290 | 0 | 3.4 | Cytoplasm |
| 1. 529 | A0A097NT54 | Uncharacterized protein | Unknown | Protein predicted | 10.17 | 13450 | 0 | 2.2 | Cytoplasm |
| 1. 51 | A0A097NSX6 | Uncharacterized protein | Unknown | Protein Predicted | 7.65 | 13820 | 0 | 6 | Cytoplasm |
| 1. 84 | A0A097NSJ0 | F0F1 ATP synthase subunit epsilon | proton-transporting ATPase activity, rotational mechanism,ATP synthesis coupled proton transport | Protein Predicted | 6.1 | 14071 | 0 | 2.5 | Cytoplasm |
| 1. 496 | A0A097NTP2 | Uncharacterized protein | Unknown | Protein predicted | 4.65 | 14504 | 0 | 2.1 | Cytoplasm |
| 1. 338 | A0A097NT63 | Cytidine deaminase (EC 3.5.4.5) | cytidine deaminase activity, zinc ion binding | Protein predicted | 6.81 | 14618 | 0 | 2.3 | Cytoplasm |
| 1. 558 | A0A097NT05 | Uncharacterized protein | Unknown | Protein predicted | 9.71 | 14899 | 0 | 5 | Cytoplasm |
| 1. 410 | A0A097NTN1 | tRNA threonylcarbamoyladenosine biosynthesis protein TsaE | threonylcarbamoyladenosine biosynthetic process | Protein predicted | 5.37 | 15475 | 0 | 2.4 | Cytoplasm |
| 1. 54 | A0A097NTZ4 | Uncharacterized protein | Unknown | Protein Predicted | 9.52 | 16036 | 0 | 3.8 | Cytoplasm |
| 1. 144 | A0A097NTB9 | 50S ribosomal protein L23 | nucleotide binding, rRNA binding, Structural constituent of ribosome, translation | Protein inferred from homology | 9.56 | 16163 | 0 | 5 | Cytoplasm |
| 1. 71 | A0A097NSK8 | Uncharacterized protein | Unknown | Protein Predicted | 9.6 | 16445 | 0 | 4.1 | Cytoplasm |
| 1. 37 | A0A097NSH7 | Uncharacterized protein | Unknown | Protein Predicted | 9.9 | 16450 | 0 | 2.5 | Cytoplasm |
| 1. 472 | A0A097NTM6 | 50S ribosomal protein L9 | rRNA binding, Structural constituent of ribosome, translation | Protein inferred from homology | 9.4 | 16552 | 0 | 3.1 | Cytoplasm |
| 1. 483 | A0A097NSQ0 | Nudix hydrolase family protein | hydrolase activity | Protein inferred from homology | 5.22 | 16680 | 0 | 4 | Cytoplasm |
| 1. 478 | A0A097NU21 | Ribosomal RNA large subunit methyltransferase H (EC 2.1.1.177) (23S rRNA (pseudouridine1915-N3)-methyltransferase) (23S rRNA m3Psi1915 methyltransferase) (rRNA (pseudouridine-N3-)-methyltransferase RlmH) | rRNA (pseudouridine-N3-)-methyltransferase activity | Protein inferred from homology | 9.08 | 16687 | 0 | 4.6 | Cytoplasm |
| 1. 169 | A0A097NT00 | SsrA-binding protein | RNA binding | Protein inferred from homology | 9.8 | 16727 | 0 | 3.8 | Cytoplasm |
| 1. 101 | A0A097NT43 | Transcriptional regulator MraZ | DNA binding, cell division | Protein inferred from homology | 5.55 | 17008 | 0 | 3.9 | Cytoplasm |
| 1. 279 | A0A097NTM0 | Putative ribose-5-phosphate isomerase B (EC 5.3.1.6) | ribose-5-phosphate isomerase activity, carbohydrate metabolic process | Protein predicted | 6.08 | 17252 | 0 | 4.7 | Cytoplasm |
| 1. 462 | A0A097NSS0 | IscU/NifU family protein | iron ion binding, iron-sulfur cluster binding, iron-sulfur cluster assembly | Protein predicted | 8.28 | 17465 | 0 | 3.8 | Cytoplasm |
| 1. 256 | A0A097NSR0 | Transcription elongation factor GreA (Transcript cleavage factor GreA) | DNA binding, translation elongation factor activity, regulation of DNA-templated transcription, elongation, transcription, DNA-templated | Protein inferred from homologyi | 5.2 | 17546 | 0 | 3.8 | Cytoplasm |
| 1. 347 | A0A097NSR7 | Single-stranded DNA-binding protein | single-stranded DNA binding, DNA replication | Protein inferred from homologyi | 5.09 | 17724 | 0 | 2.4 | Cytoplasm |
| 1. 181 | A0A097NTQ5 | Endoribonuclease YbeY (EC 3.1.-.-) | endoribonuclease activity, metalloendopeptidase activity, zinc ion binding, rRNA processing | Protein inferred from homology | 8.83 | 17853 | 0 | 3.7 | Cytoplasm |
| 1. 553 | A0A097NTE2 | Uncharacterized protein | Unknown | Protein predicted | 5.58 | 18239 | 0 | 3.3 | Cytoplasm |
| 1. 332 | A0A097NT85 | Uncharacterized protein | Unknown | Protein predicted | 9.35 | 18281 | 0 | 4.9 | Cytoplasm |
| 1. 239 | A0A097NT78 | 50S ribosomal protein L10 | large ribosomal subunit rRNA binding, translation, ribosome biogenesis | Protein inferred from homology | 9.14 | 18311 | 0 | 2.5 | Cytoplasm |
| 1. 130 | A0A097NST6 | Transcription antitermination protein NusB | RNA binding, regulation of transcription, DNA-templated | Protein Predicted | 6.62 | 18584 | 0 | 2.7 | Cytoplasm |
| 1. 446 | A0A097NTJ7 | Adenine phosphoribosyltransferase (APRT) (EC 2.4.2.7) | adenine phosphoribosyltransferase activity, adenine salvage, AMP salvage | Protein inferred from homology | 7.76 | 18819 | 0 | 3.7 | Cytoplasm |
| 1. 509 | A0A097NTE9 | Uncharacterized protein | Unknown | Protein predicted | 9.6 | 18933 | 0 | 6.3 | Cytoplasm |
| 1. 475 | A0A097NSM3 | Large-conductance mechanosensitive channel | ion channel activity | Protein predicted | 9.26 | 19102 | 0 | 4.2 | Cytoplasm |
| 1. 539 | A0A097NU07 | Uncharacterized protein | Unknown | Protein predicted | 6 | 19191 | 0 | 3.9 | Cytoplasm |
| 1. 162 | A0A097NTC4 | Uncharacterized protein | Unknown | Protein Predicted | 6.3 | 19431 | 0 | 2.8 | Cytoplasm |
| 1. 532 | A0A097NT72 | Uncharacterized protein | Unknown | Protein predicted | 9.76 | 19513 | 4 | 5.5 | Cytoplasm |
| 1. 524 | A0A097NTT0 | Membrane protein | Unknown | Protein predicted | 10.04 | 19791 | 2 | 4.7 | Cytoplasm |
| 1. 514 | A0A097NTQ1 | Bacteriophage MHoV1 protein HtpH | Unknown | Protein predicted | 5.12 | 20231 | 0 | 3.2 | Cytoplasm |
| 1. 486 | A0A097NSX7 | Elongation factor P (EF-P) | translation elongation factor activity, peptide biosynthetic process | Protein inferred from homology | 5.36 | 20441 | 0 | 4 | Cytoplasm |
| 1. 11 | A0A097NTQ8 | Peptidyl-tRNA hydrolase (PTH) (EC 3.1.1.29) | aminoacyl-tRNA hydrolase activity, translation | Protein inferred from homology | 8.46 | 21088 | 0 | 3.6 | Cytoplasm |
| 1. 53 | A0A097NSJ1 | ATP synthase subunit delta (ATP synthase F(1) sector subunit delta) (F-type ATPase subunit delta) | proton-transporting ATP synthase activity, rotational mechanism, plasma membrane ATP synthesis coupled proton transport | Protein inferred from homology | 6.76 | 21093 | 0 | 5.3 | Cytoplasm |
| 1. 330 | A0A097NTM5 | Probable GTP-binding protein EngB | GTPase activity, GTP binding, magnesium ion binding, barrier septum assembly | Protein inferred from homologyi | 9.17 | 21129 | 0 | 4 | Cytoplasm |
| 1. 276 | A0A097NSY9 | Inorganic pyrophosphatase (EC 3.6.1.1) (Pyrophosphate phospho-hydrolase) | inorganic diphosphatase activity, magnesium ion binding, phosphate-containing compound metabolic process | Protein inferred from homologyi | 5.11 | 21183 | 0 | 4.1 | Cytoplasm |
| 1. 126 | A0A097NSS3 | Deoxycytidylate deaminase (EC 3.5.4.33) | hydrolase activity | Protein Predicted | 8.91 | 21287 | 0 | 4.3 | Cytoplasm |
| 1. 147 | A0A097NT08 | Hypoxanthine phosphoribosyltransferase (EC 2.4.2.8) | guanine phosphoribosyltransferase activity, purine ribonucleoside salvage | Protein Predicted | 7.69 | 21469 | 0 | 2.8 | Cytoplasm |
| 1. 473 | A0A097NSQ3 | Peptide deformylase (PDF) (EC 3.5.1.88) (Polypeptide deformylase) | Unknown | Protein inferred from homology | 7.79 | 21540 | 0 | 3.2 | Cytoplasm |
| 1. 352 | A0A097NSZ3 | Holliday junction ATP-dependent DNA helicase RuvA (EC 3.6.4.12) | ATP binding,DNA binding, four-way junction helicase activity, DNA recombination, DNA repair, SOS response | Protein inferred from homology | 7.7 | 21597 | 0 | 4.5 | Cytoplasm |
| 1. 327 | A0A097NSI5 | ATP synthase subunit b (ATP synthase F(0) sector subunit b) (ATPase subunit I) (F-type ATPase subunit b) | proton-transporting ATP synthase activity, rotational mechanism, plasma membrane ATP synthesis coupled proton transport | Protein inferred from homologyi | 8.98 | 21673 | 0 | 4.2 | Cytoplasm |
| 1. 556 | A0A097NSS4 | Uncharacterized protein | Unknown | Protein predicted | 6.83 | 22116 | 0 | 4.2 | Cytoplasm |
| 1. 444 | A0A097NSL3 | Thymidine kinase (EC 2.7.1.21) | ATP binding, thymidine kinase activity, zinc ion binding, DNA biosynthetic process | Protein inferred from homology | 8.5 | 22416 | 0 | 4.9 | Cytoplasm |
| 1. 198 | A0A097NSL0 | FMN-dependent NADH-azoreductase (EC 1.7.-.-) (Azo-dye reductase) (FMN-dependent NADH-azo compound oxidoreductase) | electron carrier activity, FMN reductase activity, oxidoreductase activity, acting on other nitrogenous compounds as donors, FMN binding, oxidoreductase activity, acting on NAD(P)H, NAD(P) as acceptor | Protein inferred from homology | 8.57 | 22452 | 0 | 3.6 | Cytoplasm |
| 1. 297 | A0A097NTN0 | Transcription termination/antitermination protein NusG | unreviewedDNA-templated transcription, elongation, DNA-templated transcription, termination, regulation of DNA-templated transcription, elongation, transcription antitermination | Protein inferred from homologyi | 6.01 | 22561 | 0 | 3.5 | Cytoplasm |
| 1. 507 | A0A097NTT8 | Uncharacterized protein | Unknown | Protein predicted | 5.78 | 23208 | 0 | 2.5 | Cytoplasm |
| 1. 1 | A0A097NTG2 | Uracil phosphoribosyltransferase (EC 2.4.2.9) (UMP pyrophosphorylase) (UPRTase) | GTP binding, uracil phosphoribosyltransferase activity, UMP salvage | Protein inferred from homology | 6.11 | 23,570 | 0 | 3.4 | Cytoplasm |
| 1. 123 | A0A097NTC6 | Lipoprotein signal peptidase (EC 3.4.23.36) | aspartic-type endopeptidase activity, | Protein inferred from homology | 8.44 | 23620 | 0 | 3.3 | Cytoplasm |
| 1. 85 | A0A097NSK6 | Segregation and condensation protein B | cell division | Protein inferred from homology | 4.82 | 23882 | 0 | 2.8 | Cytoplasm |
| 1. 12 | A0A097NTX8 | Guanylate kinase (EC 2.7.4.8) (GMP kinase) | ATP binding, guanylate kinase activity, purine nucleotide metabolic process | Protein inferred from homology | 6.47 | 23918 | 6.5 | 6.8 | Cytoplasm |
| 1. 155 | A0A097NTM7 | tRNA (guanine-N(7)-)-methyltransferase (EC 2.1.1.33) (tRNA (guanine(46)-N(7))-methyltransferase) (tRNA(m7G46)-methyltransferase) | tRNA (guanine-N7-)-methyltransferase activity | Protein inferred from homology | 9.17 | 23965 | 7 | 5.8 | Cytoplasm |
| 1. 399 | A0A097NTQ9 | Uncharacterized protein | Unknown | Protein predicted | 7.69 | 24114 | 0 | 5.2 | Cytoplasm |
| 1. 42 | A0A097NTG6 | Deoxyribose-phosphate aldolase (DERA) (EC 4.1.2.4) (2-deoxy-D-ribose 5-phosphate aldolase) (Phosphodeoxyriboaldolase) | deoxyribose-phosphate aldolase activity, carbohydrate catabolic process, deoxyribose phosphate catabolic process | Protein inferred from homology | 6.2 | 24311 | 0 | 4.1 | Cytoplasm |
| 1. 334 | A0A097NTU8 | Ribulose-phosphate 3-epimerase (EC 5.1.3.1) | ribulose-phosphate 3-epimerase activity, carbohydrate metabolic process | Protein predicted | 6.23 | 24757 | 0 | 3.4 | Cytoplasm |
| 1. 433 | A0A097NTN6 | Uracil-DNA glycosylase (UDG) (EC 3.2.2.27) | uracil DNA N-glycosylase activity, base-excision repair | Protein inferred from homology | 9.31 | 24785 | 0 | 3.8 | Cytoplasm |
| 1. 369 | A0A097NSP5 | 50S ribosomal protein L1 | rRNA binding, Structural constituent of ribosome, tRNA binding, regulation of translation, translation | Protein inferred from homology | 9.72 | 24957 | 0 | 5.1 | Cytoplasm |
| 1. 111 | A0A097NSK1 | Potassium transporter KtrA | cation transmembrane transporter activity, potassium ion transport | Protein Predicted | 5.39 | 25599 | 0 | 4.5 | Cytoplasm |
| 1. 319 | A0A097NT40 | Ribosomal RNA small subunit methyltransferase E (EC 2.1.1.193) | methyltransferase activity, rRNA processing | Protein inferred from homologyi | 5.99 | 25764 | 4.5 | 5.9 | Cytoplasm |
| 1. 17 | A0A097NSK0 | TrmH family tRNA/rRNA methyltransferase (EC 2.1.1.-) | RNA binding, RNA processing | Protein predicted | 9.02 | 25843 | 0 | 7.2 | Cytoplasm |
| 1. 222 | A0A097NTZ0 | Putative ABC transporter ATP-binding protein (EC 3.6.3.-) | ATPase activity, ATP binding | Protein inferred from homology | 8.96 | 26113 | 0 | 4.4 | Cytoplasm |
| 1. 236 | A0A097NSZ9 | Deoxyguanosine kinase (EC 2.7.1.113) | ATP binding,deoxyguanosine kinase activity | Protein Predicted | 5.69 | 26163 | 0 | 4.8 | Cytoplasm |
| 1. 460 | A0A097NSE0 | 5'-methylthioadenosine/S-adenosylhomocysteine nucleosidase (EC 3.2.2.9) | adenosylhomocysteine nucleosidase activity, nucleoside metabolic process | Protein predicted | 7.5 | 26327 | 0 | 6.8 | Cytoplasm |
| 1. 270 | A0A097NTG9 | Purine nucleoside phosphorylase DeoD-type (EC 2.4.2.1) | purine-nucleoside phosphorylase activity, nucleoside metabolic process | Protein predicted | 5.33 | 26434 | 0 | 5.8 | Cytoplasm |
| 1. 345 | A0A097NT99 | Adenylate kinase (AK) (EC 2.7.4.3) (ATP-AMP transphosphorylase) (ATP:AMP phosphotransferase) (Adenylate monophosphate kinase) | adenylate kinase activity, ATP binding, AMP salvage | Protein inferred from homologyi | 8.75 | 26458 | 3 | 7.5 | Cytoplasm |
| 1. 250 | A0A097NTG4 | Ribonuclease HIII (EC 3.1.26.4) | RNA binding,RNA-DNA hybrid ribonuclease activity | Protein inferred from homologyi | 9.22 | 26612 | 0 | 6.1 | Cytoplasm |
| 1. 260 | A0A097NT09 | Probable transcriptional regulatory protein yebC | unreviewedDNA binding,regulation of transcription, DNA-templated,transcription, DNA-templated | Protein inferred from homologyi | 5.05 | 26908 | 0 | 4.3 | Cytoplasm |
| 1. 67 | A0A097NT39 | Putative TrmH family tRNA/rRNA methyltransferase (EC 2.1.1.-) | RNA binding, RNA processing | Protein Predicted | 9.06 | 27123 | 0 | 3 | Cytoplasm |
| 1. 273 | A0A097NSY0 | Release factor glutamine methyltransferase (EC 2.1.1.-) | nucleic acid binding,protein methyltransferase activity | Protein predicted | 9.84 | 27444 | 4 | 6.4 | Cytoplasm |
| 1. 108 | A0A097NSW1 | Ribosomal large subunit pseudouridine synthase B (EC 5.4.99.22) | pseudouridine synthase activity, RNA binding, pseudouridine synthesis | Protein inferred from homology | 9.57 | 27566 | 0 | 4.6 | Cytoplasm |
| 1. 402 | A0A097NT82 | Methionine aminopeptidase (MAP) (MetAP) (EC 3.4.11.18) (Peptidase M) | metal ion binding, metalloaminopeptidase activity, protein initiator methionine removal | Protein inferred from homology | 6.52 | 27620 | 0 | 3.6 | Cytoplasm |
| 1. 377 | A0A097NTG0 | Ribosomal RNA small subunit methyltransferase G (EC 2.1.1.-) (16S rRNA 7-methylguanosine methyltransferase) | rRNA (guanine-N7-)-methyltransferase activity | Protein inferred from homology | 9.08 | 27712 | 0 | 5 | Cytoplasm |
| 1. 131 | A0A097NSN2 | Uncharacterized protein | Unknown | Protein Predicted | 5.2 | 27831 | 0 | 4.8 | Cytoplasm |
| 1. 226 | A0A097NSS7 | 16S/23S rRNA (Cytidine-2'-O)-methyltransferase TlyA (EC 2.1.1.226) | methyltransferase activity, RNA binding | Protein Predicted | 8.8 | 28065 | 0 | 4.3 | Cytoplasm |
| 1. 305 | A0A097NSR6 | Uncharacterized protein | Unknown | Protein predicted | 9.63 | 28253 | 0 | 4.8 | Cytoplasm |
| 1. 442 | A0A097NTY0 | Serine/threonine phosphatase Stp (EC 3.1.3.16) | phosphoprotein phosphatase activity | Protein predicted | 6.73 | 28402 | 0 | 5.5 | Cytoplasm |
| 1. 348 | A0A097NT16 | CDP-diacylglycerol--glycerol-3-phosphate 3-phosphatidyltransferase (EC 2.7.8.5) | CDP-diacylglycerol-glycerol-3-phosphate 3-phosphatidyltransferase activity, phospholipid biosynthetic process | Protein inferred from homologyi | 9.71 | 28869 | 0 | 2.2 | Cytoplasm |
| 1. 97 | A0A097NTI8 | ABC transporter ATP-binding protein | ATPase activity | Protein inferred from homology | 9.15 | 28895 | 0 | 5.8 | Cytoplasm |
| 1. 458 | A0A097NT23 | DNA processing protein DprA (SMF) | DNA mediated transformation | Protein predicted | 8.49 | 29306 | 0 | 4.4 | Cytoplasm |
| 1. 450 | A0A097NSF1 | TatD family hydrolase (EC 3.1.21.-) | endodeoxyribonuclease activity, producing 5'-phosphomonoesters | Protein predicted | 5.06 | 29430 | 0 | 2 | Cytoplasm |
| 1. 301 | A0A097NTG1 | Uncharacterized protein | Unknown | Protein predicted | 8.69 | 29764 | 0 | 4.1 | Cytoplasm |
| 1. 333 | A0A097NTI6 | Choline/ethanolamine kinase family protein LicA | unreviewedkinase activity | Protein predicted | 7.02 | 29775 | 0 | 3.7 | Cytoplasm |
| 1. 278 | A0A097NT06 | Energy-coupling factor transporter ATP-binding protein EcfA (ECF transporter A component EcfA) (EC 3.6.3.-) | ATPase activity, ATP binding, transport | Protein inferred from homologyi | 5.83 | 29784 | 0 | 3.8 | Cytoplasm |
| 1. 268 | A0A097NTP9 | COF family hydrolase | hydrolase activity | Protein predicted | 9.54 | 30138 | 0 | 4.9 | Cytoplasm |
| 1. 41 | A0A097NSV1 | Putative O-methyltransferase | methyltransferase activity | Protein Predicted | 9 | 30278 | 0 | 3.6 | Cytoplasm |
| 1. 351 | A0A097NSP4 | Metallophosphoesterase | Unknown | Protein predicted | 6.18 | 30383 | 0 | 3.2 | Cytoplasm |
| 1. 416 | A0A097NTP0 | Protein GrpE (HSP-70 cofactor) | adenyl-nucleotide exchange factor activity, protein folding | Protein inferred from homology | 5.73 | 30555 | 0 | 3.3 | Cytoplasm |
| 1. 541 | A0A097NTL2 | Uncharacterized protein | Unknown | Protein predicted | 9.69 | 31217 | 0 | 5.6 | Cytoplasm |
| 1. 40 | A0A097NT52 | Putative N(G),N(G)-dimethylarginine dimethylaminohydrolase (EC 3.5.3.18) | dimethylargininase activity | Protein Predicted | 7.46 | 31695 | 0 | 3 | Cytoplasm |
| 1. 477 | A0A097NTN8 | ABC transporter ATP-binding protein (EC 3.6.3.-) | unreviewedATPase activity, ATP binding | Protein predicted | 8.21 | 31830 | 0 | 5 | Cytoplasm |
| 1. 212 | A0A097NSN8 | Uncharacterized protein | Unknown | Protein Predicted | 9.33 | 31865 | 6 | 5.6 | Cytoplasm |
| 1. 203 | A0A097NTM1 | Formamidopyrimidine-DNA glycosylase (EC 3.2.2.23) | damaged DNA binding, oxidized purine nucleobase lesion DNA N-glycosylase activity, zinc ion binding,base-excision repair | Protein Predicted | 9.4 | 31883 | 2.5 | 4.1 | Cytoplasm |
| 1. 223 | A0A097NSJ9 | Segregation and condensation protein A | cell cycle, cell division, chromosome segregation | Protein inferred from homology | 5.11 | 31984 | 0 | 4.6 | Cytoplasm |
| 1. 355 | A0A097NSF0 | F0F1 ATP synthase subunit A | hydrogen ion transmembrane transporter activity, ATP synthesis coupled proton transport | Protein predicted | 7.84 | 32073 | 4 | 3.2 | Cytoplasm |
| 1. 207 | A0A097NSK5 | Fructose-bisphosphate aldolase (EC 4.1.2.13) | fructose-bisphosphate aldolase activity, zinc ion binding, carbohydrate metabolic process | Protein Predicted | 8.75 | 32122 | 0 | 2.9 | Cytoplasm |
| 1. 120 | A0A097NTY2 | Putative ribosome biogenesis GTPase RsgA (EC 3.6.1.-) | GTPase activity, GTP binding, metal ion binding | Protein inferred from homology | 8.98 | 32303 | 0 | 4.1 | Cytoplasm |
| 1. 380 | A0A097NTT3 | Bacteriophage MHoV1 protein HtpE | Unknown | Protein predicted | 5.78 | 32576 | 0 | 4.9 | Cytoplasm |
| 1. 35 | A0A097NTR7 | Methionyl-tRNA formyltransferase (EC 2.1.2.9) | methionyl-tRNA formyltransferase activity | Protein Predicted | 9.06 | 32579 | 2 | 5.1 | Cytoplasm |
| 1. 75 | A0A097NSR9 | DNA polymerase III subunit delta | Unknown | Protein Predicted | 8.62 | 32678 | 0 | 4 | Cytoplasm |
| 1. 300 | A0A097NSH9 | Ribosomal large subunit pseudouridine synthase C (EC 5.4.99.24) | pseudouridine synthase activity, RNA binding, pseudouridine synthesis | Protein predicted | 9.42 | 32718 | 0 | 5.8 | Cytoplasm |
| 1. 128 | A0A097NT34 | Ribosome biogenesis GTPase A | GTP binding | Protein inferred from homology | 9.57 | 32814 | 0 | 6.2 | Cytoplasm |
| 1. 27 | A0A097NT47 | tRNA pseudouridine synthase B (EC 5.4.99.25) | pseudouridine synthase activity, pseudouridine synthesis, tRNA processing | Protein Predicted | 9.32 | 32942 | 2 | 4.1 | Cytoplasm |
| 1. 145 | A0A097NSI7 | Carbamate kinase | carbamate kinase activity,arginine metabolic process | Protein inferred from homology | 6.98 | 32972 | 0 | 2.8 | Cytoplasm |
| 1. 248 | A0A097NSP1 | Ribonuclease 3 (EC 3.1.26.3) (Ribonuclease III) | metal ion binding, ribonuclease III activity, rRNA binding, mRNA processing, rRNA catabolic process, rRNA processing, tRNA processing | Protein inferred from homology | 5.81 | 33045 | 0 | 4.3 | Cytoplasm |
| 1. 193 | A0A097NSX5 | Uncharacterized protein | DNA binding | Protein Predicted | 8.53 | 33130 | 0 | 4.9 | Cytoplasm |
| 1. 22 | A0A097NT79 | DegV domain-containing protein | lipid binding | Protein predicted | 6.36 | 33329 | 0 | 4.4 | Cytoplasm |
| 1. 423 | A0A097NTZ5 | Elongation factor Ts (EF-Ts) | translation elongation factor activity | Protein inferred from homology | 5.5 | 33347 | 0 | 3.5 | Cytoplasm |
| 1. 228 | A0A097NTU6 | Spermidine/putrescine ABC transporter permease protein | transport, | Protein inferred from homology | 9.59 | 33388 | 0 | 3.8 | Cytoplasm |
| 1. 45 | A0A097NT01 | Bifunctional protein FolD | methenyltetrahydrofolate cyclohydrolase activity, folic acid-containing compound biosynthetic process, methionine biosynthetic process, tetrahydrofolate interconversion | Protein inferred from homology | 9.14 | 33845 | 0 | 4.2 | Cytoplasm |
| 1. 121 | A0A097NTH4 | Thioredoxin reductase (EC 1.8.1.9) | flavin adenine dinucleotide binding | Protein Predicted | 5.92 | 33853 | 0 | 5.1 | Cytoplasm |
| 1. 210 | A0A097NTW5 | Putative kinase (EC 2.7.1.-) | diacylglycerol kinase activity, protein kinase C-activating G-protein coupled receptor signaling pathway | Protein Predicted | 9.49 | 34004 | 0 | 5.2 | Cytoplasm |
| 1. 312 | A0A097NSV8 | Pseudouridine synthase | Responsible for synthesis of pseudouridine from uracil, pseudouridine synthase activity | Protein inferred from homology | 8.56 | 34,146 | 0 | 5.2 | Cytoplasm |
| 1. 109 | A0A097NTU1 | Bacteriophage MHoV1 replication protein RepB (EC 3.6.4.12) | ATP binding, DNA replication | Protein Predicted | 5.36 | 34355 | 0 | 4.5 | Cytoplasm |
| 1. 9 | A0A097NTP5 | 5'-3' exonuclease (EC 2.7.7.7) | DNA binding, DNA-directed DNA polymerase activity, exonuclease activity | protein predicted | 6.23 | 34374 | 0 | 3.9 | Cytoplasm |
| 1. 267 | A0A097NTV4 | Nucleotidyltransferase family protein | transferase activity | Protein predicted | 8.78 | 34844 | 0 | 5.6 | Cytoplasm |
| 1. 425 | A0A097NTL5 | Uncharacterized protein | Unknown | Protein predicted | 9.22 | 35033 | 0 | 4.3 | Cytoplasm |
| 1. 229 | A0A097NTL1 | Membrane protein | Unknown | Protein Predicted | 9.8 | 35078 | 4 | 4.1 | Cytoplasm |
| 1. 206 | A0A097NTH9 | Cof family hydrolase (EC 3.1.3.-) | hydrolase activity | Protein Predicted | 5.25 | 35230 | 0 | 2.7 | Cytoplasm |
| 1. 459 | A0A097NTM9 | tRNA N6-adenosine threonylcarbamoyltransferase (EC 2.6.99.4) (N6-L-threonylcarbamoyladenine synthase) (t(6)A37 threonylcarbamoyladenosine biosynthesis protein TsaD) (tRNA threonylcarbamoyladenosine biosynthesis protein TsaD) | iron ion binding, metalloendopeptidase activity, transferase activity, transferring acyl groups other than amino-acyl groups, threonylcarbamoyladenosine biosynthetic process | Protein inferred from homology | 8.46 | 35280 | 0 | 4.6 | Cytoplasm |
| 1. 269 | A0A097NTE5 | Uncharacterized protein | Unknown | Protein predicted | 9.24 | 35493 | 0 | 6 | Cytoplasm |
| 1. 538 | A0A097NTP8 | DnaB-related protein | Unknown | Protein predicted | 5.29 | 35799 | 10.5 | 7.6 | Cytoplasm |
| 1. 5 | A0A097NT14 | Cobalt ABC transporter permease | Unknown | protein Predicted | 9.85 | 35998 | 5 | 4.4 | Cytoplasm |
| 1. 157 | A0A097NTE3 | COF family hydrolase | hydrolase activity | Protein Predicted | 6.04 | 36053 | 4 | 5.8 | Cytoplasm |
| 1. 4 | A0A097NTI9 | Ribose-phosphate pyrophosphokinase (EC 2.7.6.1) | kinase activity, ribose phosphate diphosphokinase activity, nucleotide biosynthetic process | Protein predicted | 8.19 | 36072 | 0 | 4.2 | Cytoplasm |
| 1. 289 | A0A097NSZ2 | Holliday junction ATP-dependent DNA helicase RuvB (EC 3.6.4.12) | ATP binding, DNA binding, four-way junction helicase activity, DNA recombination, DNA repair, SOS response | Protein inferred from homologyi | 9.13 | 36093 | 4 | 6.2 | Cytoplasm |
| 1. 13 | A0A097NU10 | Proline iminopeptidase (EC 3.4.11.5) | aminopeptidase activity | Protein inferred from homology | 5.67 | 36112 | 0 | 3.3 | Cytoplasm |
| 1. 56 | A0A097NSQ2 | Uncharacterized protein | Unknown | Protein Predicted | 4.88 | 36374 | 0 | 3.4 | Cytoplasm |
| 1. 421 | A0A097NSS2 | Phenylalanyl-tRNA synthetase subunit alpha (EC 6.1.1.20) | ATP binding, phenylalanine-tRNA ligase activity, tRNA binding, phenylalanyl-tRNA aminoacylation | Protein inferred from homology | 5.13 | 36391 | 0 | 2.7 | Cytoplasm |
| 1. 316 | A0A097NSR4 | Protein RecA (Recombinase A) | ATP binding, damaged DNA binding, DNA-dependent ATPase activity, single-stranded DNA binding, DNA recombination, DNA repair,SOS response | Protein inferred from homologyi | 7.68 | 36734 | 0 | 3.5 | Cytoplasm |
| 1. 150 | A0A097NTX4 | Glyceraldehyde-3-phosphate dehydrogenase (EC 1.2.1.12) | glyceraldehyde-3-phosphate dehydrogenase (NAD+) (phosphorylating) activity, NAD binding, glucose metabolic process | Protein inferred from homology | 6.68 | 36790 | 0 | 4.9 | Cytoplasm |
| 1. 463 | A0A097NTI2 | ABC transporter ATP-binding protein | ATPase activity, ATP binding | Protein inferred from homology | 9.33 | 36806 | 0 | 5.6 | Cytoplasm |
| 1. 482 | A0A097NSU8 | Uncharacterized protein | Unknown | Protein predicted | 5.45 | 36963 | 0 | 3.8 | Cytoplasm |
| 1. 487 | A0A097NT24 | Lipoprotein | Unknown | Protein predicted | 8.89 | 37193 | 0 | 4.5 | Cytoplasm |
| 1. 76 | A0A097NSN0 | Tryptophan--tRNA ligase (EC 6.1.1.2) (Tryptophanyl-tRNA synthetase) | ATP binding, tryptophanyl-tRNA aminoacylation | Protein inferred from homology | 8.92 | 37265 | 0 | 7.2 | Cytoplasm |
| 1. 187 | A0A097NU22 | DHH family phosphoesterase (EC 3.1.-.-) | hydrolase activity, nucleic acid binding | Protein Predicted | 5.74 | 37429 | 0 | 4.5 | Cytoplasm |
| 1. 140 | A0A097NTX1 | Phosphate acyltransferase (EC 2.3.1.n2) (Acyl-ACP phosphotransacylase) (Acyl-[acyl-carrier-protein]--phosphate acyltransferase) (Phosphate-acyl-ACP acyltransferase) | oxidoreductase activity, acting on the CH-OH group of donors, NAD or NADP as acceptor, transferase activity, transferring acyl groups other than amino-acyl groups,fatty acid biosynthetic process, phospholipid biosynthetic process | Protein inferred from homology | 8.41 | 37498 | 0 | 6.9 | Cytoplasm |
| 1. 264 | A0A097NT19 | DNA polymerase III subunit delta | DNA binding, DNA-directed DNA polymerase activity, DNA replication | Protein predicted | 5 | 37545 | 0 | 3.1 | Cytoplasm |
| 1. 434 | A0A097NTY9 | DHH family phosphoesterase (EC 3.1.-.-) | hydrolase activity | Protein predicted | 6.27 | 37670 | 0 | 4.5 | Cytoplasm |
| 1. 96 | A0A097NTK0 | Aspartate--ammonia ligase (EC 6.3.1.1) | aminoacyl-tRNA ligase activity, ATP binding, L-asparagine biosynthetic process | Protein inferred from homology | 7.15 | 37723 | 0 | 7.8 | Cytoplasm |
| 1. 468 | A0A097NU27 | Nicotinate phosphoribosyltransferase (EC 6.3.4.21) | ligase activity, nicotinate-nucleotide diphosphorylase (carboxylating) activity, NAD biosynthetic process | Protein predicted | 7.69 | 37931 | 2.5 | 3.7 | Cytoplasm |
| 1. 65 | A0A097NSM4 | Ornithine carbamoyltransferase, catabolic (EC 2.1.3.3) | amino acid binding, ornithine carbamoyltransferase activity | Protein inferred from homology | 6.2 | 38319 | 0 | 3.9 | Cytoplasm |
| 1. 354 | A0A097NTU5 | Serine/threonine-protein kinase PrkC (EC 2.7.11.1) | ATP binding, protein serine/threonine kinase activity | Protein predicted | 8.99 | 38675 | 0 | 4.6 | Cytoplasm |
| 1. 246 | A0A097NT76 | DNA-directed RNA polymerase subunit alpha (RNAP subunit alpha) (EC 2.7.7.6) (RNA polymerase subunit alpha) (Transcriptase subunit alpha) | DNA binding, DNA-directed RNA polymerase activity, transcription, DNA-templated | Protein inferred from homology | 5.36 | 38953 | 0 | 6.8 | Cytoplasm |
| 1. 295 | A0A097NTV8 | Signal recognition particle receptor FtsY (SRP receptor) | GTPase activity, GTP binding, SRP-dependent cotranslational protein targeting to membrane | Protein inferred from homologyi | 6.42 | 39118 | 0 | 4.1 | Cytoplasm |
| 1. 527 | A0A097NTH5 | Membrane protein | Unknown | Protein predicted | 6.04 | 39141 | 0 | 3.5 | Cytoplasm |
| 1. 280 | A0A097NT20 | Xaa-Pro aminopeptidase (EC 3.4.-.-) | aminopeptidase activity,metalloexopeptidase activity | Protein predicted | 5.34 | 39162 | 0 | 4.7 | Cytoplasm |
| 1. 88 | A0A097NT04 | Energy-coupling factor transporter ATP-binding protein EcfA (ECF transporter A component EcfA) (EC 3.6.3.-) | ATPase activity, transport | Protein inferred from homology | 9.1 | 39268 | 6 | 7.1 | Cytoplasm |
| 1. 233 | A0A097NTK4 | M42 glutamyl aminopeptidase family protein | aminopeptidase activity | Protein Predicted | 5.5 | 39990 | 0 | 5.8 | Cytoplasm |
| 1. 313 | A0A097NTP4 | Heat-inducible transcription repressor HrcA | DNA binding, negative regulation of transcription, DNA-templated, transcription, DNA-templated | Protein inferred from homologyi | 6.99 | 40021 | 0 | 6.2 | Cytoplasm |
| 1. 63 | A0A097NTP6 | Bacteriophage MHoV1 integrase | DNA binding, DNA integration | Protein Predicted | 8.91 | 40599 | 0 | 5.6 | Cytoplasm |
| 1. 234 | A0A097NT65 | Alanine racemase-like protein (EC 5.1.1.1) | alanine racemase activity | Protein Predicted | 8.17 | 40729 | 0 | 4.7 | Cytoplasm |
| 1. 424 | A0A097NT46 | Cell division protein FtsZ | GTPase activity, GTP binding, barrier septum assembly, FtsZ-dependent cytokinesis, protein polymerization | Protein inferred from homology | 4.73 | 40781 | 2 | 2.7 | Cytoplasm |
| 1. 464 | A0A097NSW5 | Peptide chain release factor 1 (RF-1) | translation release factor activity, codon specific | Protein inferred from homology | 5.06 | 40903 | 0 | 6.5 | Cytoplasm |
| 1. 15 | A0A097NTD6 | AAA ATPase family protein (EC 3.4.24.-) | ATP binding | Protein predicted | 5.83 | 40956 | 0 | 3.9 | Cytoplasm |
| 1. 326 | A0A097NSD5 | DNA polymerase III subunit beta (EC 2.7.7.7) | 3'-5' exonuclease activity, DNA binding, DNA-directed DNA polymerase activity, DNA replication | Protein predicted | 5.06 | 41347 | 0 | 5 | Cytoplasm |
| 1. 91 | A0A097NSH5 | Carboxypeptidase related protein (EC 3.4.16.-) | carboxypeptidase activity | Protein Predicted | 8.46 | 41525 | 0 | 6.6 | Cytoplasm |
| 1. 80 | A0A097NSL2 | Uncharacterized protein | Unknown | Protein Predicted | 9.79 | 41827 | 4 | 5.3 | Cytoplasm |
| 1. 467 | A0A097NT77 | Uncharacterized protein | sequence-specific DNA binding | Protein predicted | 7.23 | 41942 | 0 | 6.1 | Cytoplasm |
| 1. 143 | A0A097NSU1 | Nicotinate-nucleotide adenylyltransferase (EC 2.7.7.18) | ATP binding, NAD biosynthetic process | Protein inferred from homology | 9.15 | 42147 | 0 | 3.7 | Cytoplasm |
| 1. 500 | A0A097NTT2 | Bacteriophage MHoV1 protein HtpTb | Unknown | Protein predicted | 8.62 | 42199 | 0 | 4 | Cytoplasm |
| 1. 244 | A0A097NSQ4 | tRNA-specific 2-thiouridylase MnmA (EC 2.8.1.-) | ATP binding, sulfurtransferase activity, tRNA binding, tRNA modification | Protein inferred from homology | 8.32 | 42393 | 0 | 3.5 | Cytoplasm |
| 1. 356 | A0A097NSW0 | Chaperone protein DnaJ | ATP binding, zinc ion binding, DNA replication, protein folding, response to heat | Protein inferred from homology | 7.53 | 42502 | 2 | 5 | Cytoplasm |
| 1. 177 | A0A097NTH1 | S-adenosylmethionine synthase (AdoMet synthase) (EC 2.5.1.6) (MAT) (Methionine adenosyltransferase) | ATP binding, methionine adenosyltransferase activity, one-carbon metabolic process | Protein inferred from homology | 5.38 | 42881 | 0 | 4.6 | Cytoplasm |
| 1. 146 | A0A097NSI9 | Elongation factor Tu (EF-Tu) | GTPase activity, GTP binding, translation elongation factor activity, | Protein inferred from homology | 5.75 | 43596 | 0 | 3.1 | Cytoplasm |
| 1. 394 | A0A097NSS8 | Oligopeptide transport ATP-binding protein OppD | ATPase activity,ATP binding, peptide transporter activity | Protein predicted | 9.06 | 43832 | 2 | 3.8 | Cytoplasm |
| 1. 216 | A0A097NSY5 | Alcohol dehydrogenase (EC 1.1.1.-) | metal ion binding,oxidoreductase activity | Protein Predicted | 6.48 | 43850 | 0 | 6.4 | Cytoplasm |
| 1. 129 | A0A097NSK9 | Phosphoglycerate kinase (EC 2.7.2.3) | ATP binding, phosphoglycerate kinase activity, glycolytic process | Protein inferred from homology | 6.94 | 43955 | 0 | 4.5 | Cytoplasm |
| 1. 211 | A0A097NSU4 | Aminotransferase class V (EC 2.8.1.7) | cysteine desulfurase activity, pyridoxal phosphate binding, transaminase activity | Protein inferred from homology | 5.87 | 44004 | 0 | 4.6 | Cytoplasm |
| 1. 298 | A0A097NTK7 | Probable tRNA sulfurtransferase (EC 2.8.1.4) (Sulfur carrier protein ThiS sulfurtransferase) (Thiamine biosynthesis protein ThiI) (tRNA 4-thiouridine synthase) | ATP binding, sulfurtransferase activity, tRNA adenylyltransferase activity, tRNA binding, thiamine biosynthetic process, thiamine diphosphate biosynthetic process,tRNA thio-modification | Protein inferred from homologyi | 8.58 | 44046 | 0 | 4.7 | Cytoplasm |
| 1. 534 | A0A097NTT6 | Bacteriophage MHoV1 protein HtpTa | Unknown | Protein predicted | 4.66 | 44271 | 0 | 3.8 | Cytoplasm |
| 1. 158 | A0A097NTM3 | Phosphopentomutase (EC 5.4.2.7) (Phosphodeoxyribomutase) | magnesium ion binding,phosphopentomutase activity, 5-phosphoribose 1-diphosphate biosynthetic process, deoxyribonucleotide catabolic process | Protein inferred from homology | 6.2 | 44420 | 0 | 2.7 | Cytoplasm |
| 1. 142 | A0A097NSV3 | Putative ABC transporter permease protein | transport | Protein inferred from homology | 9.35 | 45604 | 0 | 5.4 | Cytoplasm |
| 1. 561 | A0A097NTT7 | Bacteriophage MHoV1 protein HtpN | Unknown | Protein predicted | 5.08 | 45860 | 0 | 3.6 | Cytoplasm |
| 1. 8 | A0A097NTF3 | Type I restriction enzyme specificity protein | DNA binding, DNA modification | protein predicted | 8.01 | 46198 | 0 | 5.7 | Cytoplasm |
| 1. 479 | A0A097NST1 | Replication-associated recombination protein A | DNA binding, four-way junction helicase acivity, DNA recombination, DNA repair, DNA replication | Protein predicted | 6.47 | 46280 | 0 | 6.4 | Cytoplasm |
| 1. 367 | A0A097NSM9 | Arginine deiminase (ADI) (EC 3.5.3.6) (Arginine dihydrolase) | arginine deiminase activity, arginine catabolic process to ornithine | Protein inferred from homology | 5.52 | 46289 | 0 | 6.6 | Cytoplasm |
| 1. 397 | A0A097NTW7 | Serine hydroxymethyltransferase (SHMT) (Serine methylase) (EC 2.1.2.1) | glycine hydroxymethyltransferase activity, methyltransferase activity, pyridoxal phosphate binding, glycine biosynthetic process from serine, tetrahydrofolate interconversion | Protein inferred from homology | 7.14 | 46525 | 4.5 | 7.6 | Cytoplasm |
| 1. 141 | A0A097NSM7 | Cysteine--tRNA ligase (EC 6.1.1.16) (Cysteinyl-tRNA synthetase) | ATP binding, cysteine-tRNA ligase activity, zinc ion binding, cysteinyl-tRNA aminoacylation | Protein inferred from homology | 7.63 | 46788 | 0 | 4.1 | Cytoplasm |
| 1. 274 | A0A097NSV9 | GTPase Obg (GTP-binding protein Obg) | GTPase activity, GTP binding, magnesium ion binding | Protein inferred from homologyi | 5.55 | 46904 | 0 | 4.5 | Cytoplasm |
| 1. 72 | A0A097NTH7 | Uncharacterized protein | Unknown | Protein Predicted | 9.2 | 47050 | 2 | 9.7 | Cytoplasm |
| 1. 518 | A0A097NSS9 | Uncharacterized protein | Unknown | Protein predicted | 9.35 | 47072 | 0 | 6.7 | Cytoplasm |
| 1. 230 | A0A097NT68 | Tyrosine--tRNA ligase (EC 6.1.1.1) (Tyrosyl-tRNA synthetase) | ATP binding, RNA binding, tyrosine-tRNA ligase activity, tyrosyl-tRNA aminoacylation | Protein inferred from homology | 7.02 | 47137 | 0 | 4.3 | Cytoplasm |
| 1. 205 | A0A097NSU9 | Oligopeptide transport system permease protein OppC | transport, | Protein inferred from homology | 9.4 | 47238 | 0 | 4.4 | Cytoplasm |
| 1. 58 | A0A097NTE4 | Pyrimidine-nucleoside phosphorylase (EC 2.4.2.2) | phosphorylase activity, pyrimidine nucleobase metabolic process | Protein Predicted | 7.61 | 47634 | 0 | 4.4 | Cytoplasm |
| 1. 95 | A0A097NTD7 | DNA modification methylase (EC 2.1.1.37) | DNA (cytosine-5-)-methyltransferase activity, DNA restriction-modification system | Protein inferred from homology | 9 | 48064 | 0 | 5.5 | Cytoplasm |
| 1. 469 | A0A097NTQ4 | Serine--tRNA ligase (EC 6.1.1.11) (Seryl-tRNA synthetase) (Seryl-tRNA(Ser/Sec) synthetase) | ATP binding, serine-tRNA ligase activity, selenocysteine biosynthetic process, selenocysteinyl-tRNA(Sec) biosynthetic process, seryl-tRNA aminoacylation | Protein inferred from homology | 6.5 | 48202 | 0 | 6.5 | Cytoplasm |
| 1. 195 | A0A097NSK3 | Glutamyl-tRNA(Gln) amidotransferase subunit A (EC 6.3.5.-) | carbon-nitrogen ligase activity, with glutamine as amido-N-donor | Protein Predicted | 6.05 | 48701 | 5.5 | 5.9 | Cytoplasm |
| 1. 432 | A0A097NU01 | Putative dipeptidase (EC 3.4.13.-) | dipeptidase activity, metallopeptidase activity | Protein predicted | 5.47 | 49462 | 0 | 5 | Cytoplasm |
| 1. 173 | A0A097NSN4 | Uncharacterized protein | Unknown | Protein Predicted | 5.35 | 49641 | 0 | 5.3 | Cytoplasm |
| 1. 471 | A0A097NTR9 | tRNA modification GTPase MnmE (EC 3.6.-.-) | GTPase activity, GTP binding, metal ion binding, tRNA modification | Protein inferred from homology | 6.3 | 49803 | 0 | 4.7 | Cytoplasm |
| 1. 163 | A0A097NT28 | Signal recognition particle protein (Fifty-four homolog) | 7S RNA binding, GTP binding, SRP-dependent cotranslational protein targeting to membrane | Protein inferred from homology | 9.27 | 49982 | 0 | 5.5 | Cytoplasm |
| 1. 350 | A0A097NSX1 | GTPase Der (GTP-binding protein EngA) | GTP binding, ribosome biogenesis, small GTPase mediated signal transduction | Protein inferred from homology | 8.71 | 49985 | 0 | 5.3 | Cytoplasm |
| 1. 31 | A0A097NSZ6 | Histidine--tRNA ligase (EC 6.1.1.21) (Histidyl-tRNA synthetase) | ATP binding, histidyl-tRNA aminoacylation | Protein inferred from homology | 6.63 | 50109 | 0 | 8 | Cytoplasm |
| 1. 47 | A0A097NSQ7 | Enolase (EC 4.2.1.11) (2-phospho-D-glycerate hydro-lyase) (2-phosphoglycerate dehydratase) | magnesium ion binding, glycolytic process | Protein inferred from homology | 6.25 | 50431 | 0 | 4.9 | Cytoplasm |
| 1. 73 | A0A097NTN2 | Cytosol aminopeptidase (EC 3.4.11.1) | aminopeptidase activity, metalloexopeptidase activity | Protein Predicted | 6.37 | 50715 | 0 | 5 | Cytoplasm |
| 1. 60 | A0A097NTG7 | Glucose-6-phosphate isomerase (EC 5.3.1.9) | glucose-6-phosphate isomerase activity, gluconeogenesis | Protein Predicted | 6.43 | 50729 | 0 | 6 | Cytoplasm |
| 1. 465 | A0A097NTR6 | Cytosol aminopeptidase (EC 3.4.11.1) | aminopeptidase activity, manganese ion binding, metalloexopeptidase activity | Protein predicted | 5.14 | 50903 | 0 | 4.6 | Cytoplasm |
| 1. 235 | A0A097NTC0 | F0F1 ATP synthase subunit beta (EC 3.6.3.14) | ATP binding, proton-transporting ATP synthase activity, rotational mechanism, ATP hydrolysis coupled proton transport, ATP synthesis coupled proton transport | Protein Predicted | 5.57 | 50975 | 0 | 5.1 | Cytoplasm |
| 1. 94 | A0A097NTR5 | Asparagine--tRNA ligase (EC 6.1.1.22) (Asparaginyl-tRNA synthetase) | asparagine-tRNA ligase activity, ATP binding, nucleic acid binding, asparaginyl-tRNA aminoacylation, | Protein inferred from homology | ??? | 51303 | 0 | 4.9 | Cytoplasm |
| 1. 186 | A0A097NTV7 | Putative RNA uracil-methyltransferase (EC 2.1.1.189) | RNA binding,RNA processing | Protein Predicted | 8.75 | 51456 | 0 | 4.9 | Cytoplasm |
| 1. 184 | A0A097NSH4 | Aminopeptidase C (EC 3.4.22.40) | aminopeptidase activity, cysteine-type endopeptidase activity | Protein Predicted | 5.33 | 51552 | 0 | 5.2 | Cytoplasm |
| 1. 152 | A0A097NTS1 | Membrane protein | Unknown | Protein Predicted | 9.47 | 52660 | 0 | 2.9 | Cytoplasm |
| 1. 62 | A0A097NTJ6 | Replicative DNA helicase (EC 3.6.4.12) | ATP binding, DNA helicase activity, DNA replication | Protein Predicted | 5.86 | 52814 | 0 | 4.6 | Cytoplasm |
| 1. 224 | A0A097NSE7 | Chromosomal replication initiator protein DnaA | ATP binding, sequence-specific DNA binding, DNA replication initiation | Protein inferred from homology | 7.63 | 52961 | 0 | 5.5 | Cytoplasm |
| 1. 10 | A0A097NTQ0 | Pyruvate kinase (EC 2.7.1.40) | magnesium ion binding, pyruvate kinase activity, glycolytic process | Protein inferred from homology | 7.66 | 53011 | 0 | 5.1 | Cytoplasm |
| 1. 168 | A0A097NT25 | Glycyl-tRNA synthetase (EC 6.1.1.14) | ATP binding, glycyl-tRNA aminoacylation | Protein inferred from homology | 6.37 | 53589 | 0 | 3.5 | Cytoplasm |
| 1. 294 | A0A097NTE0 | Type II restriction enzyme (EC 3.1.21.4) | DNA binding, Type II site-specific deoxyribonuclease activity | Protein predicted | 9 | 53958 | 0 | 4 | Cytoplasm |
| 1. 287 | A0A097NTQ3 | Proline--tRNA ligase (EC 6.1.1.15) (Prolyl-tRNA synthetase) | ATP binding, proline-tRNA ligase activity, prolyl-tRNA aminoacylation | Protein inferred from homologyi | 6.41 | 54168 | 0 | 4.7 | Cytoplasm |
| 1. 520 | A0A097NTD9 | Uncharacterized protein | Unknown | Protein predicted | 9.06 | 54440 | 0 | 4.6 | Cytoplasm |
| 1. 92 | A0A097NTP7 | Bacteriophage MHoV1 protein HtpA | Unknown | Protein Predicted | 10.26 | 54629 | 2 | 6.3 | Cytoplasm |
| 1. 522 | A0A097NTF9 | Uncharacterized protein | Unknown | Protein predicted | 9.02 | 54670 | 0 | 4.6 | Cytoplasm |
| 1. 337 | A0A097NSI8 | ATP synthase subunit beta (EC 3.6.3.14) (ATP synthase F1 sector subunit beta) (F-ATPase subunit beta) | ATP binding, proton-transporting ATP synthase activity, rotational mechanism, ATP hydrolysis coupled proton transport, plasma membrane ATP synthesis coupled proton transport | Protein inferred from homologyi | 5.71 | 54694 | 0 | 4.4 | Cytoplasm |
| 1. 376 | A0A097NSK4 | Aspartyl/glutamyl-tRNA(Asn/Gln) amidotransferase subunit B (Asp/Glu-ADT subunit B) (EC 6.3.5.-) | ATP binding, glutaminyl-tRNA synthase (glutamine-hydrolyzing) activity, transferase activity, translation | Protein inferred from homology | 6.12 | 54812 | 0 | 3.9 | Cytoplasm |
| 1. 149 | A0A097NSL5 | Trigger factor (TF) (EC 5.2.1.8) (PPIase) | peptidyl-prolyl cis-trans isomerase activity, cell cycle, protein folding | Protein inferred from homology | 5.22 | 54812 | 3 | 4.5 | Cytoplasm |
| 1. 112 | A0A097NT87 | Protein translocase subunit SecY | intracellular protein transmembrane transport, protein transport by the Sec complex | Protein inferred from homology | 9.51 | 54926 | 6 | 3.9 | Cytoplasm |
| 1. 540 | A0A097NT69 | Uncharacterized protein | Unknown | Protein predicted | 8.91 | 55374 | 0 | 5.1 | Cytoplasm |
| 1. 429 | A0A097NSU5 | Uncharacterized protein | Unknown | Protein predicted | 8.95 | 55844 | 0 | 7.2 | Cytoplasm |
| 1. 331 | A0A097NTN5 | Lysine--tRNA ligase (EC 6.1.1.6) (Lysyl-tRNA synthetase) | ATP binding, lysine-tRNA ligase activity, magnesium ion binding, nucleic acid binding, lysyl-tRNA aminoacylation | Protein inferred from homologyi | 5.67 | 56790 | 0 | 3.4 | Cytoplasm |
| 1. 308 | A0A097NTE8 | Uncharacterized protein | Unknown | Protein predicted | 9.9 | 57701 | 0 | 5 | Cytoplasm |
| 1. 176 | A0A097NTE1 | F0F1 ATP synthase subunit alpha (EC 3.6.3.14) | ATP binding, proton-transporting ATP synthase activity, rotational mechanism, ATP hydrolysis coupled proton transport | Protein Predicted | 6.84 | 57927 | 3.5 | 5.3 | Cytoplasm |
| 1. 148 | A0A097NSG6 | ATP synthase subunit alpha (EC 3.6.3.14) (ATP synthase F1 sector subunit alpha) (F-ATPase subunit alpha) | ATP binding, proton-transporting ATPase activity, rotational mechanism, proton-transporting ATP synthase activity, rotational mechanism, ATP hydrolysis coupled proton transport, plasma membrane ATP synthesis coupled proton transport | Protein inferred from homology | 6 | 58393 | 0 | 3.7 | Cytoplasm |
| 1. 115 | A0A097NTD3 | Type I restriction modification system DNA methyltransferase HsdM (EC 2.1.1.72) | DNA binding, site-specific DNA-methyltransferase (adenine-specific) activity, DNA restriction-modification system | Protein Predicted | 4.96 | 58863 | 0 | 3.8 | Cytoplasm |
| 1. 172 | A0A097NU11 | Type I restriction modification system DNA methyltransferase HsdM (EC 2.1.1.72) | DNA binding, site-specific DNA-methyltransferase (adenine-specific) activity,DNA restriction-modification system | Protein Predicted | 4.96 | 58921 | 0 | 3.8 | Cytoplasm |
| 1. 225 | A0A097NTW9 | Fatty acid kinase ATP-binding subunit FakA | ATP binding, glycerone kinase activity, glycerol metabolic process | Protein Predicted | 5.15 | 59058 | 0 | 4.7 | Cytoplasm |
| 1. 202 | A0A097NTK8 | Uncharacterized protein | transferase activity, transferring phosphorus-containing groups | Protein Predicted | 8.81 | 59725 | 0 | 5.8 | Cytoplasm |
| 1. 328 | A0A097NTF1 | ABC transporter ATP-binding protein | ATPase activity, ATP binding | Protein predicted | 5.43 | 61366 | 0 | 4.8 | Cytoplasm |
| 1. 428 | A0A097NSU2 | Methionyl-tRNA synthetase (EC 6.1.1.10) | ATP binding, methionine-tRNA ligase activity, methionyl-tRNA aminoacylation | Protein predicted | 7.62 | 61396 | 0 | 6.1 | Cytoplasm |
| 1. 362 | A0A097NSL8 | Arginine--tRNA ligase (EC 6.1.1.19) (Arginyl-tRNA synthetase) | arginine-tRNA ligase activity,ATP binding,arginyl-tRNA aminoacylation | Protein inferred from homology | 6.27 | 61490 | 0 | 6.8 | Cytoplasm |
| 1. 3 | A0A097NU16 | Transcription termination/antitermination protein NusA | RNA binding, regulation of DNA-templated transcription, termination | Protein inferred from homology | 4.74 | 62427 | 0 | 4.3 | Cytoplasm |
| 1. 322 | A0A097NTK2 | mRNA degradation ribonucleases J2 (EC 3.1.-.-) | endonuclease activity, RNA binding,metal ion binding | Protein predicted | 8.89 | 62598 | 5 | 7 | Cytoplasm |
| 1. 404 | A0A097NTW4 | Putative serine/threonine exchanger SteT | amino acid transmembrane transporter activity | Protein predicted | 8.88 | 63285 | 0 | 3.6 | Cytoplasm |
| 1. 214 | A0A097NTX6 | MATE family protein | antiporter activity,drug transmembrane transporter activity | Protein Predicted | 9.03 | 64972 | 0 | 3.2 | Cytoplasm |
| 1. 103 | A0A097NSZ7 | Aspartate--tRNA ligase (EC 6.1.1.12) (Aspartyl-tRNA synthetase) | aspartate-tRNA ligase activity, nucleic acid binding, aspartyl-tRNA aminoacylation | Protein inferred from homology | 6.42 | 65406 | 0 | 2.4 | Cytoplasm |
| 1. 55 | A0A097NTH2 | mRNA degradation ribonucleases J1 (EC 3.1.-.-) | endonuclease activity, RNA binding | Protein inferred from homology | 8.81 | 65536 | 0 | 4.3 | Cytoplasm |
| 1. 448 | A0A097NSF6 | DNA methylase | DNA binding, N-methyltransferase activity, DNA methylation | Protein predicted | 5.6 | 65554 | 0 | 6.1 | Cytoplasm |
| 1. 515 | A0A097NTQ6 | Bacteriophage MHoV1 protein MarRP | Unknown | Protein predicted | 7.09 | 66970 | 4 | 3.5 | Cytoplasm |
| 1. 381 | A0A097NU15 | Translation initiation factor IF-2 | GTPase activity, GTP binding, translation initiation factor activity, | Protein inferred from homology | 5.9 | 67568 | 0 | 5.7 | Cytoplasm |
| 1. 119 | A0A097NTP3 | Elongation factor 4 (EF-4) (EC 3.6.5.n1) (Ribosomal back-translocase LepA) | GTPase activity, ribosome binding, positive regulation of translation | Protein inferred from homology | 5.45 | 68110 | 3 | 6.2 | Cytoplasm |
| 1. 30 | A0A097NSP8 | Threonine--tRNA ligase (EC 6.1.1.3) (Threonyl-tRNA synthetase) | ATP binding, threonine-tRNA ligase activity, threonyl-tRNA aminoacylation | Protein inferred from homology | 7.24 | 68230 | 0 | 6.7 | Cytoplasm |
| 1. 48 | A0A097NTY6 | tRNA uridine 5-carboxymethylaminomethyl modification enzyme MnmG (Glucose-inhibited division protein A) | flavin adenine dinucleotide binding, tRNA wobble uridine modification | Protein inferred from homology | 6.71 | 68275 | 0 | 7.2 | Cytoplasm |
| 1. 61 | A0A097NSE5 | MATE family membrane protein | antiporter activity | Protein Predicted | 9.24 | 68575 | 0 | 5.1 | Cytoplasm |
| 1. 307 | A0A097NTJ1 | ABC transporter permease/ATP-binding subunits | ATPase activity, coupled to transmembrane movement of substances, ATP binding | Protein predicted | 8.76 | 70009 | 2.5 | 4.7 | Cytoplasm |
| 1. 100 | A0A097NTX0 | DNA topoisomerase IV subunit B (EC 5.99.1.3) | ATP binding, DNA topoisomerase type II (ATP-hydrolyzing) activity, DNA topological change | Protein inferred from homology | 6.62 | 71735 | 3 | 4.5 | Cytoplasm |
| 1. 242 | A0A097NTH6 | Oligoendopeptidase F (EC 3.4.24.-) | metalloendopeptidase activity | Protein Predicted | 6.79 | 71846 | 0 | 6.2 | Cytoplasm |
| 1. 153 | A0A097NT22 | DNA primase (EC 2.7.7.-) | DNA binding, zinc ion binding | Protein inferred from homology | 8.43 | 72477 | 6.5 | 4.4 | Cytoplasm |
| 1. 191 | A0A097NTL0 | DNA gyrase subunit B (EC 5.99.1.3) | ATP binding, DNA topoisomerase type II (ATP-hydrolyzing) activity, DNA-dependent DNA replication | Protein inferred from homology | 5.65 | 72626 | 0 | 4.9 | Cytoplasm |
| 1. 156 | A0A097NT57 | ClpB related chaperone protein | ATP binding | Protein Predicted | 5.61 | 74068 | 0 | 6.3 | Cytoplasm |
| 1. 215 | A0A097NTU0 | UvrABC system protein B (Protein UvrB) (Excinuclease ABC subunit B) | ATP binding, excinuclease ABC activity, DNA binding, helicase activity,nucleotide-excision repair | Protein inferred from homology | 6.61 | 75771 | 0 | 4 | Cytoplasm |
| 1. 457 | A0A097NSG2 | Membrane protein insertase YidC | protein insertion into membrane | Protein inferred from homology | 9.73 | 75895 | 0 | 5.8 | Cytoplasm |
| 1. 78 | A0A097NT07 | DNA ligase (EC 6.5.1.2) (Polydeoxyribonucleotide synthase [NAD(+)]) | DNA ligase (NAD+) activity, metal ion binding, DNA repair, DNA replication | Protein inferred from homology | 5.97 | 76271 | 0 | 4.6 | Cytoplasm |
| 1. 251 | A0A097NSN7 | DNA polymerase III subunit gamma/tau (EC 2.7.7.7) | ATP binding, DNA binding, DNA-directed DNA polymerase activity, DNA replication | Protein predicted | 5.22 | 76572 | 0 | 4.7 | Cytoplasm |
| 1. 102 | A0A097NSV2 | Elongation factor G (EF-G) | GTPase activity, translation elongation factor activity | Protein inferred from homology | 5.37 | 77982 | 0 | 7 | Cytoplasm |
| 1. 340 | A0A097NSI6 | ATP-dependent chaperone protein ClpB | ATP binding | Protein inferred from homologyi | 5.82 | 81034 | 0 | 5 | Cytoplasm |
| 1. 392 | A0A097NTQ7 | ATP-dependent zinc metalloprotease FtsH (EC 3.4.24.-) | ATPase activity, ATP binding, metalloendopeptidase activity, zinc ion binding, protein catabolic process | Protein inferred from homology | 6.1 | 81348 | 4 | 6.2 | Cytoplasm |
| 1. 271 | A0A097NTI4 | Membrane protein P80 | Unknown | Protein predicted | 8.81 | 81864 | 7 | 5.8 | Cytoplasm |
| 1. 430 | A0A097NTX9 | Transcription accessory protein Tex | nucleic acid binding, nucleobase-containing compound metabolic process | Protein predicted | 6.9 | 81929 | 0 | 5.2 | Cytoplasm |
| 1. 299 | A0A097NST9 | Phenylalanine--tRNA ligase beta subunit (EC 6.1.1.20) (Phenylalanyl-tRNA synthetase beta subunit) | ATP binding, phenylalanine-tRNA ligase activity, magnesium ion binding, tRNA binding, phenylalanyl-tRNA aminoacylation | Protein inferred from homologyi | 6.51 | 82357 | 0 | 5.6 | Cytoplasm |
| 1. 194 | A0A097NSZ8 | Ribonuclease R (RNase R) (EC 3.1.13.1) | exoribonuclease II activity | Protein inferred from homology | 7.52 | 82711 | 0 | 5.6 | Cytoplasm |
| 1. 253 | A0A097NSY3 | Uncharacterized protein | Unknown | Protein predicted | 5.93 | 82714 | 0 | 4.9 | Cytoplasm |
| 1. 302 | A0A097NTT1 | Bacteriophage MHoV1 protein HtpB | Unknown | Protein predicted | 5.4 | 82828 | 0 | 3.9 | Cytoplasm |
| 1. 44 | A0A097NT21 | Uncharacterized protein | Unknown | Protein Predicted | 8.51 | 83975 | 0 | 6.7 | Cytoplasm |
| 1. 255 | A0A097NTB8 | Peptidase S8 domain protein | serine-type endopeptidase activity | Protein predicted | 8.58 | 85590 | 0 | 5.7 | Cytoplasm |
| 1. 388 | A0A097NTM2 | RecD/TraA family helicase (EC 3.6.4.12) | helicase activity | Protein predicted | 5.74 | 86300 | 0 | 4.8 | Cytoplasm |
| 1. 443 | A0A097NTR4 | ATP-dependent DNA helicase PcrA (EC 3.6.4.12) | ATP binding, ATP-dependent DNA helicase activity, DNA binding | Protein predicted | 5.81 | 86942 | 0 | 8 | Cytoplasm |
| 1. 390 | A0A097NTA7 | Xylulose-5-phosphate phosphoketolase (EC 4.1.2.9) | phosphoketolase activity,carbohydrate metabolic process | Protein predicted | 5.91 | 90769 | 0 | 5.5 | Cytoplasm |
| 1. 257 | A0A097NTB2 | Leucine--tRNA ligase (EC 6.1.1.4) (Leucyl-tRNA synthetase) | aminoacyl-tRNA editing activity, leucine-tRNA ligase activity,ATP binding, eucyl-tRNA aminoacylation | Protein inferred from homologyi | 6.43 | 93019 | 0 | 5 | Cytoplasm |
| 1. 113 | A0A097NTL4 | ATP-dependent protease Lon (EC 3.4.21.53) | ATP binding, ATP-dependent peptidase activity, serine-type endopeptidase activity, protein catabolic process | Protein inferred from homology | 5.77 | 93624 | 0 | 6.1 | Cytoplasm |
| 1. 263 | A0A097NU13 | Type I restriction modification system specificity subunit HsdS | DNA binding,DNA modification | Protein predicted | 9.1 | 93830 | 0 | 6.5 | Cytoplasm |
| 1. 398 | A0A097NSI1 | Type III restriction enzyme, res subunit | ATP binding, DNA binding, hydrolase activity | Protein predicted | 6.54 | 95627 | 0 | 3.9 | Cytoplasm |
| 1. 57 | A0A097NSX0 | Oligopeptide transport ATP-binding protein OppF | ATPase activity, peptide transport | Protein inferred from homology | 8.99 | 98793 | 0 | 6 | Cytoplasm |
| 1. 70 | A0A097NSQ6 | Protein translocase subunit SecA | ATP binding, intracellular protein transmembrane transport, protein targeting | Protein inferred from homology | 5.26 | 99403 | 0 | 7.3 | Cytoplasm |
| 1. 117 | A0A097NSU6 | Alanine--tRNA ligase (EC 6.1.1.7) (Alanyl-tRNA synthetase) | alanine-tRNA ligase activity, tRNA binding, alanyl-tRNA aminoacylation | Protein inferred from homology | 5.66 | 102489 | 0 | 5.2 | Cytoplasm |
| 1. 190 | A0A097NTD0 | Isoleucine--tRNA ligase (EC 6.1.1.5) (Isoleucyl-tRNA synthetase) | aminoacyl-tRNA editing activity, ATP binding, isoleucine-tRNA ligase activity, zinc ion binding,isoleucyl-tRNA aminoacylation | Protein inferred from homology | 6.03 | 102962 | 2 | 5.2 | Cytoplasm |
| 1. 231 | A0A097NTG5 | Magnesium-transporting ATPase (EC 3.6.3.2) | ATP binding, magnesium-importing ATPase activity, metal ion binding | Protein inferred from homology | 8.48 | 104451 | 9 | 5.1 | Cytoplasm |
| 1. 220 | A0A097NTH3 | UvrABC system protein A | Excision nuclease | Protein inferred from homology | 7.91 | 105723 | 3.5 | 5 | Cytoplasm |
| 1. 375 | A0A097NTZ8 | Cation transporting P-type ATPase (EC 3.6.3.8) | ATP binding,calcium-transporting ATPase activity,metal ion binding | Protein inferred from homology | 8.56 | 107148 | 2 | 6.1 | Cytoplasm |
| 1. 49 | A0A097NU04 | DNA topoisomerase IV subunit A (EC 5.99.1.3) | ATP binding, DNA topoisomerase type II (ATP-hydrolyzing) activity, DNA topological change | Protein Predicted | 6.14 | 107420 | 0 | 5 | Cytoplasm |
| 1. 218 | A0A097NTY8 | Uncharacterized protein | Unknown | Protein Predicted | 8.77 | 116224 | 3 | 7.3 | Cytoplasm |
| 1. 445 | A0A097NTY1 | Type I restriction modification system endonuclease subunit HsdR (EC 3.1.21.3) | ATP binding, Type I site-specific deoxyribonuclease activity, DNA restriction-modification system | Protein predicted | 6.89 | 120143 | 2 | 4.5 | Cytoplasm |
| 1. 209 | A0A097NT70 | DNA-directed RNA polymerase subunit beta (RNAP subunit beta) (EC 2.7.7.6) (RNA polymerase subunit beta) (Transcriptase subunit beta) | DNA binding, DNA-directed RNA polymerase activity, ribonucleoside binding, transcription, DNA-templated | Protein inferred from homology | 5.64 | 134943 | 3 | 4.9 | Cytoplasm |
| 1. 407 | A0A097NSR2 | ATP-binding protein | ATP binding | Protein predicted | 4.88 | 163409 | 3 | 6.4 | Cytoplasm |
| 1. 28 | A0A097NTW2 | DNA polymerase III PolC-type (PolIII) (EC 2.7.7.7) | 3'-5' exonuclease activity, DNA-directed DNA polymerase activity, DNA-dependent DNA replication | Protein inferred from homology | 6.37 | 165512 | 0 | 6.2 | Cytoplasm |
| 1. 237 | A0A097NT86 | DNA-directed RNA polymerase subunit beta' (RNA polymerase subunit beta') (Transcriptase subunit beta') | DNA binding, DNA-directed RNA polymerase activity, transcription, DNA-templated | Protein inferred from homology | 5.83 | 165886 | 0 | 5.4 | Cytoplasm |
| 1. 323 | A0A097NT44 | Uncharacterized protein | Unknown | Protein predicted | 5.58 | 183892 | 0 | 6.2 | Cytoplasm |
| 1. 175 | A0A097NTM4 | Membrane protein | Unknown | Protein Predicted | 9.5 | 552115 | 10.5 | 4.3 | Cytoplasm |
|  | A0A097NT12 | Putative glucan 1,6-alpha-glucosidase | carbohydrate metabolic process | Protein Predicted | 8.94 | 58167 | 11.5 | 3.4 | Cytoplasm |
